# A worldwide geographical scheme for recording the distribution of marine biota: proposal and call for feedback

**DOI:** 10.3897/BDJ.13.e157371

**Published:** 2025-07-31

**Authors:** Nicolas Bailly, Serge Gofas, Britt Lonneville

**Affiliations:** 1 University of British Columbia / Beaty Biodiversity Museum, Vancouver, Canada University of British Columbia / Beaty Biodiversity Museum Vancouver Canada; 2 FishBase/SeaLifeBase, Q quatics, Manila, Philippines FishBase/SeaLifeBase, Q quatics Manila Philippines; 3 University of Malaga, Malaga, Spain University of Malaga Malaga Spain; 4 Muséum National d'Histoire Naturelle, Paris, France Muséum National d'Histoire Naturelle Paris France; 5 Marine Regions, Flanders Marine Institute, Oostende, Belgium Marine Regions, Flanders Marine Institute Oostende Belgium

**Keywords:** taxonomic databases, checklists, marine distributions, marine biota, biodiversity data, marine bioregions

## Abstract

This paper describes a project aimed at creating a worldwide set of polygons for recording marine distribution data, parallel to the current World Geographic Scheme for Recording Plant Distribution used on land. The countries’ Exclusive Economic Zones were either taken as recording units or subdivided according to Marine Ecosystems of the World or the IHO Limits of Oceans and Seas when appropriate; existing local schemes were adopted for Europe and Australia. A hierarchical set of five Level-1 units, 26 Level-2 units, 232 Level-3 units and 536 Level-4 units is presented for feedback and intended to be submitted as a standard to the Biodiversity Information Standards (TDWG). This project is expected to provide a means to instantly retrieve national checklists for any taxonomic group and also a valuable tool to handle imprecise country-level records from the old literature.

## Introduction

More than thirty years ago, in the early stage of the Internet, the International Working Group on Taxonomic Databases (TDWG) recognised the need for an agreed system of geographical units at approximately “country” level and upwards for use in recording plant distributions. The deliberations over a period of three years of a committee involving taxonomists as well as applied botanists culminated in producing a standard World Geographic Scheme for Recording Plant Distributions (hereafter WGSRPD; Hollis and Brummitt (1992); Brummitt et al. (2001)) which covers the whole world and has been widely accepted. The scheme is now used in major databases. for example, The World Flora Online Consortium (2024) and hundreds of scientific papers (over 600 citations in Google Scholar), not only botanical.

With an increasing wealth of data available in the marine realm, a similar standard is desirable for the sea. Geo-referenced data are accurately represented in such databases as the Ocean Biodiversity Information System 2000 (see Grassle (2000)) and the Global Biodiversity Information Facility 2001 (see Gaiji et al. (2013)), but there is also a need for pre-defined geographical units as a tool for the purpose of: (1) classification and retrieval of distribution data, be they expressed as areas or as point data and (2) handling data formulated as the name of a country or region, with no more detail and no coordinates. We believe that, like it was agreed on for the terrestrial system, these geounits should be politically bound in order to meet the needs of end-users.

With respect to land, two peculiarities of the world ocean make major differences. Firstly, it is a three-dimensional realm where the water column can span thousands of metres and harbours a vertical succession of thoroughly different biotic communities. The vertical distribution of these communities is driven by factors such as light penetration and the characteristics of water masses. In addition, the communities settled on the sea bottom (benthos) are radically different from those of the water column (plankton and nekton). On a map, those cannot be discriminated unless depth is added to geography. The other major difference with the terrestrial realm is that only ca. 42% of the world ocean surface is under the jurisdiction of coastal states, whereas the remaining 58% are so far “high seas” where there are no political boundaries. Therefore, the rationale of using political boundaries must be complemented by decisions regarding the treatment of “high seas”.

The present proposal is intended to parallel as much as possible the well-established terrestrial scheme, about which Hollis and Brummitt (1992) wrote: “WGSRPD provides an agreed system of geographical units at approximately "country" level and upwards for use in recording plant distributions. It allows adopting organizations to compare and exchange data with each other without loss of information due to incompatible geographical boundaries. (...) Very large countries, however, have been subdivided into more conveniently sized units according to constituent states or provinces”.

Our endeavour is not starting from scratch. A wealth of marine (and also land) geographical units is stored, with associated descriptions and shapefiles, on the Marine Regions website (Flanders Marine Institute 2012, hereafter VLIZ, for its Flemish acronym). These units are classified according to “placetypes”, which are sets of geographical units established from the same source and/or with the same rationale. One of these “placetypes” is precisely the terrestrial WGSRPD scheme and others with relevance to marine areas will be mentioned hereafter where appropriate.

In this paper, we review existing sets of geounits defined in the marine realm and discuss their suitability for the stated purpose, explain the rationale we intend to apply to the marine scheme and put forward a proposal for a comprehensive set of marine geounits, with also four levels of hierarchy. Following TDWG procedures, this proposal is posted on a GitHub repository where it is publicly accessible and open to comments. The main reason for presenting this communication is that, before implementing a marine equivalent of WGSRPD, the proposal would have better chances to be widely accepted if it is first exposed to the scrutiny and feedback from a scientific community interested in biogeography and conservation policy.

## State of the art: Existing sets of geounits in the marine realm

In this section, we discuss some of the existing schemes for subdivision of the marine realm and their suitability as potential recording units. Most of these are also listed by Sayre et al. (2017) (Table 1) and Zhao et al. (2020) (Table 1). Some schemes are exclusively political (e.g. EEZ, MSFD), geographical (e.g. IHO, GOODS), whilst others have a more or less explicitly biogeographical background. An extended version, with illustrations of these existing schemes, is presented as supplementary material (Suppl. material 1) also posted on the project’s GitHub repository by TDWG Geoschemes Task Group (2022) and is here summarised.

### IHO – The International Hydrographic Organization

The International Hydrographic Organization has published a detailed geographical scheme for subdivision of the world ocean (International Hydrographic Organization 1953) into named areas, for which shapefiles are stored on the MarineRegions website. The largest IHO polygons could be candidate geounits for Level 1 of a universal marine scheme if they encompassed the entire oceans, but this is not the case as many parts of oceans and seas are named as separate geounits of the same level (e.g. Gulf of Guinea and Bay of Biscay are not part of the North Atlantic Ocean, but adjacent units of the same rank).

### Exclusive Economic Zones (EEZs) and the “Marine Region” placetype at VLIZ

The EEZs are jurisdictional waters declared by coastal states under the provisions of the United Nations Convention for the Law of the Sea (UNCLOS). For those states which have not declared so far an EEZ, the UNCLOS considers the concept of a “continental shelf” (not constrained to the physiographic continental shelf) which is granted with no need for a declaration and comprises the sea-bottom to the same extent of 200 nautical miles. In the Marine Regions website, “EEZ” is taken in the broad sense of a potential EEZ from shore to a distance of 200 NM and a complete set of shapefiles is publicly available (latest version 12 October 2023).

For many small or medium-sized coastal countries and territories, the corresponding EEZ provides a suitable polygon for a basic recording unit at Level 4. The EEZs of larger countries (e.g. the United States, South Africa, Indonesia, Japan) and/or countries with a complex maritime facade (e.g. Spain, Egypt, Costa Rica) must be split.

The Marine Regions website at VLIZ has developed a set of geounits with placetype “Marine Regions” (not to be confused with the name of the website as a whole) which is the intersection of EEZs with the IHO sea areas. These are straightforward for splitting some countries with two maritime facades (e.g. Costa Rica), but there are issues which preclude universally using this set of geounits when we need to subdivide EEZs:

(1) as a consequence of IHO severing adjacent sea areas from larger oceans or seas, some EEZ/IHO intersections result in awkward representations (e.g. the “Egyptian part of the Red Sea” in Marine Regions misses Gulf of Suez and Gulf of Aqaba, deemed to be different bodies of equal rank to “Red Sea” in the IHO Scheme).

(2) in other cases, the intersection of EEZ with major Oceans in the IHO scheme are too large (e.g. United States part of the Atlantic Ocean, spanning from Maine to Florida), therefore inadequate as basic recording units.

Under certain conditions, countries apply for an “Extended Continental Shelf” beyond the 200 miles, but no further from the baseline than either 350 miles or 100 miles beyond the 2500 m isobath (whichever is more favourable; see Wu et al. (2013)); this field is currently in a state of flux and is not taken into account in the present project.

### Marine Ecoregions of the World (MEOW) of Spalding et al. (2007)

Supported by the publication of Spalding et al. (2007), this scheme aims to provide a biogeographic classification of the world's coastal and continental shelf waters following a nested hierarchy of 12 realms, 62 provinces and 232 ecoregions (with an average surface of 685.345 km^2^). This scheme has been extensively used in all kinds of global analyses (over 4000 citations in Google Scholar) and is certainly influential. Three principles were followed: that it should: (i) have a strong biogeographic basis, (ii) offer practical utility and (iii) minimise discrepancies with existing systems. At first glance, MEOWs provide units with a size largely suitable as “Basic Recording Units” (Level 4) for the coastal regions.

Nevertheless, their main drawback is that they ignore country boundaries and must therefore be adapted (intersecting a MEOW with an EEZ) if a country-based system is wanted. In this way, MEOW ecoregions are instrumental as a consistent criterion for subdividing large EEZs (e.g. Brazil, United States, Japan). At a higher hierarchical level, the MEOW Realms are also a consistent source for the definition of Level 2 units (Level 1 being the Oceans).

### Regional schemes

Coastal countries belonging to the European Union have commitments towards achieving a “Good Environmental Status” of their jurisdictional waters, as detailed in the Marine Strategies Framework Directive. This generates a need for environmental monitoring and elaboration of a suite of 11 “descriptors” (Lyons et al. 2010) and the MSFD regions were defined as a framework for reporting (Jensen et al. 2017). Countries with a composite EEZ spanning two or more of these regions (Sweden, Germany, France, Spain, Italy, Greece) have divided their jurisdictional waters into subregions according to this scheme. These administrative boundaries are highly suitable as “Basic Recording Units” and will be used in this proposal. For Spain, these polygons have been already used for the establishment of an official reference list of all marine organisms present in the country, issued in 2017 (but unfortunately withdrawn in 2020).

Another well-established regional scheme is Australia’s “Integrated Marine and Coastal Regionalisation of Australia” (IMCRA 4.0). There are only seven Level 4 units (the states) in the terrestrial WGSRPD scheme, but the marine realm is complex. No less than 18 MEOW are defined in Australian waters (Spalding et al. 2007) and 41 regions in the marine IMCRA 4.0 (Commonwealth of Australia 2006), which departs from an EEZ-based scheme by considering separately the shallow coastal regions and the remainder of the EEZ. However, it has the advantage of being a coherent system whose outer boundaries are exactly coincident with Australian EEZ. The designation of six marine regions (North, Coral Sea, Temperate East, South-east, South-west and North-west), defined in support of the Environment Protection and Biodiversity Conservation Act 1999, was supposedly (Evans et al. 2016) based on the provincial bioregions identified as part of the IMCRA 4.0, but the boundaries are not coincident, the “transition zones” recognised in the IMCRA scheme being arbitrarily split; the scheme even departs from traditional Australian marine biogeographical regions (Poore and O.'Hara 2007). The important point is to respect the contrast between temperate and tropical parts.

IMCRA regions are used for display in the Australian Faunal Directory (Australian Biological Resources Study (ABRS) 2020), whereas the Atlas of Living Australia (Belbin et al. 2021) uses only point data for the display on species factsheets, but uses the IMCRA regions as bounding box for search within an area.

A further reason for adopting the IMCRA regions as Level 4 units is that, despite their number, their extension (average surface ca. 244,000 km^2^) is similar to that of Level 4 units in many other parts of the World.

### IUCN bioregions

A two-level scheme was established by Kelleher et al. (1995) with 18 bioregions and 136 “Biogeographic zones” for the purpose of assessing the geographical representativeness of Marine Protected Areas (MPAs). It is noteworthy that IUCN itself does not use these geounits in the maps representing the geographic range of the species assessed in the Red List, but instead uses a customised representation of the distribution of coastal species on a width of less than half the extension of the EEZ. A few articles on biogeographical topics (e.g. Swart et al. (2018)) nevertheless used these IUCN Biogeographic zones. They are also used for the display of distributions in the invasive species database NEMESIS (Smithsonian Environmental Research Center 2003) although this is not explicitly stated. Drawbacks are the same as for the MEOW realms and ecoregions, with the aggravating circumstance that they are far less well-known; therefore, they will not be considered further.

### Longhurst pelagic provinces

The development of remote sensing technology makes it possible to evaluate the concentration of chlorophyll, based on the solar radiation reflected by the oceans, this being “Ocean-Colour Radiometry” (OCR), for example, the Sea-viewing Wide Field-of-view sensor (SeaWiFS) project (Hooker and McClain 2000). The relationship between chlorophyll and biomass is not fixed, but depends on the taxonomic composition of the plankton components and, therefore, on the region: hence the need for a partition of the world ocean into areas where this relationship is stable. In this perspective, Longhurst (1998) recognised four main biomes at a first level: Polar, Western, Trade Wind and Coastal biome, recognisable in all ocean basins. At a second level, each basin is divided into provinces that provide a model for data analysis, for example, assigning the necessary parameters to calculate biomass from OCR data. With their very specific focus on the open water, Longhurst provinces are not suitable as recording units on a global scale and will not be considered further.

### Large marine ecosystems

The Large Marine Ecosystem (LME) approach is aimed at improving the management of ecosystem goods and services. *Large Marine Ecosystems* (Sherman 1991) are adjacent to continents in coastal waters where primary productivity is generally higher than in the open ocean. The physical extent of the LME and its boundaries are based on four linked ecological criteria, rather than political or economic ones. These are: (i) bathymetry, (ii) hydrography, (iii) productivity and (iv) trophic relationships. Based on these four ecological criteria, 64 different LMEs have been delineated around the Atlantic, Pacific and Indian coastal margins.

### FAO Major Fishing Areas for Statistical Purposes

These are arbitrary areas (FAO Coordinating Working Party on Fishery Statistics 2025), the boundaries of which were determined in consultation with international fishery agencies and which are designed to coincide, as far as possible, with the areas of competence of fishery commissions. A total of 19 major marine fishing areas have been internationally established to date, covering the waters of the Atlantic, Indian, Pacific and Southern Oceans with their adjacent seas, being used since the 1950s for reporting the global fisheries landings.

These areas span both EEZs and high seas and their limits are not systematically coincident with political borders so that, both because of this and because of their coarse resolution, we do not find them appropriate for a more fine-grained recording of distributions.

### Global biogeographic schemes

Global regionalisations of coastal waters are based on the analysis of actual distributions of marine species, usually those belonging to well-known groups such as fishes, which reliably cover the entire marine realm. Briggs (1974) and Briggs and Bowen (2012) ranked regions at the highest hierarchical level and recognised provinces (mapped in Toonen et al. (2016)) based on an arbitrary threshold of 10% endemic species (of fish, in their data).

The marine bioregions designed by Watling and Gerken (1999) in the context of the Ocean Biodiversity Information System (OBIS) are no longer visible on their original website, but follow basically the views of Briggs (1974) which are sensible.

#### Data-driven regionalisation schemes

With the increasing amount of Geo-referenced data contained in the Ocean Biodiversity Information System (OBIS) and the Global Biodiversity Information Facility (GBIF), attempts are being made for the categorisation of biogeographic realms, based on the statistical analysis of the distribution of occurrence data worldwide, without any a-priori grouping (e.g. Costello et al. (2017), Neubauer et al. (2025)). However, such regionalisation schemes have limitations (discussed at length in Neubauer et al. (2025)) derived mostly from the utterly unequal coverage of taxa and areas in the occurrence databases (Kennedy and Rotjan 2023). Costello et al. (2017), based on the distribution of 65,000 species of marine animals and plants, both benthic and pelagic, found 30 distinct marine realms, but some of them span both high seas and coastal areas and none is coincident with the 200 NM used in the political subdivision. Therefore, even if we acknowledge the potential of such analyses, we find it premature for their integration in an essentially pragmatic set of polygons designed for the purpose of recording and retrieving species distributions.

### GOODS and Watling et al. (2023) regions for the deep sea

As mentioned earlier, 58% of the marine realm does not belong to “jurisdictional waters” and cannot be treated with the same rationale as the coastal areas. Under auspices of the Intergovernmental Oceanographic Commission (IOC) of UNESCO, the GOODS initiative presented (UNESCO 2009) a biogeographic classification for global open ocean and deep-sea areas compiled by an international expert group at a workshop held in Mexico City, Mexico, in January 2007. This was refined and formalised in a later paper by Watling et al. (2013), with of 14 lower bathyal, 14 abyssal and 10 hadal provinces. The shapefiles for the GOODS system are available on the Marine Regions website.

Watling et al. (2013) noted that most of the upper bathyal (300-800 m) is within national EEZs and considered separately the lower bathyal (plotted as 801–3500 m), the abyssal (3500–6500 m) which comprises 65.4% of the world’s seafloor and the hadal (ultra-abyssal) areas (> 6500 m). A major issue is raised because the GOODS system uses physiographic boundaries, essentially the isobaths, and largely overlaps with the 200-miles defined coastal regions. Therefore, the GOODS geounits, in order to fit the general TDWG scheme, should be intersected with the EEZs so as to prune the overlapping parts.

From a pragmatic point of view, if major oceans are taken as Level 1 in the proposed scheme, “High Seas” of each ocean can be Level 2 geounits and the Watling et al. provinces (constrained to the high seas part) incorporated in Level 3 and Level 4.

A sensible way to subdivide the large abyssal provinces of Watling et al. (2013) is using physiographic basins, largely used in some geological (e.g. Emery et al. (1975)) and taxonomic works (e.g. Allen (2008)), in which cases the Watling et al. provinces may be used at Level 3 and the individual seafloor features and basins at Level 4.

A geomorphic features map (GSFM) of the global ocean was elaborated by Harris et al. (2014), but this research focuses on the differences between active and passive margins, not on regionalisation as such. Statistics regarding undersea features are presented for each of the major oceans, but features are not named nor considered individually, therefore not bringing any helpful element for defining recording units at Level 4.

#### Regionalisation in the pelagic realm

For the pelagic realm, the difference between bathyal, abyssal and hadal is irrelevant to near-surface plankton. The mesopelagic biome was tackled by Sutton et al. (2017), but there, with the same rationale already put forward for the coastal units, preference should be given to a single set of geounits without taking habitats into account. Spalding et al. (2012) indeed produced a coherent regionalisation of the pelagic realm, but their units would conflict with those accounting for the benthos. Actually, most biogeographic data regarding plankton originate from oceanographic cruises in which samples are documented with coordinates, therefore a practical solution could be to use only higher level (Level 1 or 2) geounits as bounding boxes and point data as the basis for analyses. We acknowledge that our proposed scheme is not fitted for handling occurrences within the water column and its water masses. We still find it useful for recording such occurrences within EEZs (thereby providing information for country checklists), but for the high seas, the only sensible solution would be to define alternative Levels 3 and 4 geounits for the water column.

## Materials and methods

### TDWG Interest Groups and Task Groups

TDWG (renamed Biodiversity Information Standards in 2006, but conserving the acronym which stands for “Taxonomic Databases Working Group”) is a distributed non-profit organisation, founded in 1985, with the mission to develop, adopt and promote standards and guidelines for the recording and exchange of data about organisms. Governance is assumed by an Executive Committee consisting of ten officers elected from and by the membership of the group. One of the means to achieve its mission is to promote the use of standards (give a definition) in several categories amongst which “data standards” for content of controlled vocabularies. A well-known standard from TDWG is DarwinCore for the interoperability of species occurrence data (Wieczorek et al. 2012). Github is the main collaboration platform (adopted in 2014) and used to host and version the standards.

The adoption of TDWG standards goes through an elaborate process, which begins by the creation of an “Interest Group”, later consolidated as a “Task Group”, documented by a “charter” that defines the convenor (first contact), core members, motivation, guidance about how to become involved and a summary of what standard or guideline the group wants to achieve. The Geoschemes Interest group was approved in February 2021 by the TDWG Executive Committee and the respective Task Groups Geoschemes: Marine domain and Terrestrial domain (the update of WGSRPD) in November 2023. The corresponding charters are posted on the GitHub repository (Marine charter and Terrestrial charter).

Following section 3.3.3.1. of TDWG Standards Documentation Specification (Baskauf et al. 2017), the full name of the proposed standard must be in English and must be unique within the scope of TDWG standards. It is here proposed as **Geoschemes – marine**, as a companion standard to Geoschemes – terrestrial which is an update bound to replace the current WGSRPD.

### Rationale for the definition of units

The terrestrial WGSRPD (second edition, Brummitt et al. (2001)) has nine Level-1, 91 Level-2 , 379 Level-3 and 609 Level-4 units (will be 614 in the Geoschemes – terrestrial update currently elaborated) covering the World. This corresponds to an average surface of ca. 250,000 km^2^ for units at Level 4. As mandatory in a hierarchical system, the borders of more comprehensive levels must coincide with those of included units, with no overlaps nor discrepancies. In Geoschemes – marine, Level 1 is proposed at the scale of the major Oceans. At the lowest level, a system designed to parallel the terrestrial system should always recognise the marine jurisdictional waters of one country, however small, as a unit, but should split those of large and/or complex countries into as many units as found desirable (e.g. Costa Rica, Guatemala, albeit small, should have one Pacific and one Caribbean unit; South Africa, Brazil or China which have continuous, but very extensive coastlines spanning faunal breaks should be split).

The development of Level 2 units is not straightforward. Global biogeographic schemes (e.g. Toonen et al. (2016)) are helpful for coastal units, but are not unanimously agreed on. The most important drawback is that small, remote islands with high rates of endemism, which we would recognise at Level 4, are treated in such schemes as self-standing provinces (i.e. at the rank of Level 2 units) on the basis of quantitative differences. The same caveat applies also to data-driven partitions, where the same endemicity rates may define units of very diverse size. Therefore, these schemes will not serve the purpose of establishing a higher hierarchy. We propose to rely on the Realms recognised in the MEOW system of Spalding et al. (2007), but qualified. Firstly, those realms (Temperate South America, Temperate South Africa, Temperate Australasia) which span two of our Level 1 oceans are split accordingly. Second, in the Tropical Atlantic, we will distinguish at Level 2 between Eastern and Western Atlantic. As explained above, the MEOW system ignores political boundaries, for which reason our Level 2 coastal units will be adjusted to the nearest EEZ boundary. The high seas of each ocean will be additional Level 2 units.

Taking inspiration from the terrestrial system, Level 3 may accommodate groups of Level 4 units which share political, biogeographical and/or geographical features (e.g. the Lesser Antilles in the Caribbean Sea, each island being a Level 4 unit; the Red Sea, Black Sea, Baltic Sea, Persian Gulf), but may be coincident with Level 4 units when no obvious grouping is suggested. Reasons for grouping at Level 3 include:

(1) Level 4 units belong to a single country and are situated within the same Level 2 unit;

(2) Level 4 units which are comprised in a clearcut geographical context.

For the marine coastal Level 4 units, “coastal” is understood as the 200 NM EEZ or potential EEZ, regardless of the extension of the physical continental shelf, regardless of non-claimed or partly claimed EEZs (e.g. Greece). Claims to an “extended continental shelf” in the sense of UNCLOS, which are currently in a state of flux, are not considered at this stage. Like in the WGSRPD, no attempt is made to represent separately any particular habitats (e.g. the rocky shore, coastal marshes, the bathyal level, the water column). Admittedly, it can be found shocking to represent the distribution of a limpet which is strictly coastal as a 200 miles fringe; however, we concluded that the advantages of using distinct sets of recording units for organisms living in different compartments (e.g. intertidal vs. subtidal, pelagic vs. benthic) are by far outweighed by the advantage of having a universal scheme. Our final consideration is that, if a species is reported in the “Basic Recording Unit”, it is understood to occur “somewhere” therein, not “everywhere” therein.

Level 4 units never encompass the jurisdictional waters of more than one country or territory. For those countries with large and/or complex jurisdictional waters, these are systematically split into as many units as there are Level 1 or Level 2 units involved (e.g. Costa Rica, Guatemala, albeit small, has one Pacific and one Caribbean unit; South Africa has one Atlantic and two Indian Ocean units). In addition to this, some large EEZs (e.g. Brazil, Indonesia) are subdivided even if they are entirely contained in the same Level 2 unit. A threshold for making subdivisions is met when such EEZs involve more than one of the ecoregions in the MEOW system of Spalding et al. (2007) and then, the MEOW ecoregion boundaries are used as the template for splitting. The average surface of the 435 proposed coastal geounits is ca. 363,000 km^2^, in the same order of magnitude as the 5° cells (about 556 × 553 km or 300,000 km^2^ at the Equator) used in data-driven analyses (for example Costello et al. 2017) and somewhat larger than the terrestrial Level 4 units in WGSRPD (but many insular countries have small land surface and large EEZs).

Exceptions are made in places where there is a well-established local system of geounits with associated shapefiles: for Europe, the subregions defined for the Marine Strategy Framework Directive (MSFD; Jensen et al. (2017)); for Australia, the IMCRA regions (Commonwealth of Australia 2006). Furthermore, an ad-hoc subdivision, based on a published source, was used for places where the MEOW ecoregions have been declared inadequate (e.g. Madagascar, Cooke et al. (2023)).

We strived to design Level 4 units to match pre-existing schemes as far as possible (EEZ, IHO/EEZ intersection, MEOW). The rationale can be summarised as follows:

(1) EEZ is used wherever appropriate: not too large and contained within a single sea area [e.g. Belgium, Guinea, Uruguay];

(2) EEZ intersection with IHO is used as next choice if it has no issues [e.g. Costa Rica Caribbean and Pacific parts], using “Marine Regions” placetype (i.e. EEZ/IHO intersection) of VLIZ;

(3) if both EEZ and EEZ/IHO intersection has issues (units unwantedly split down by IHO [e.g. Egypt where Gulf of Suez and Gulf of Aqaba are not included in the IHO definition of Red Sea] or the resulting units still too large [e.g. United States part of the North Atlantic, spanning from Maine to Florida]), the EEZ is subdivided as follows:

- If an existing scheme is in usage and its units meet our requirements (no more than one country, no more than one sea area, not too large), it is adopted to subdivide the EEZ (this applies to the EU with MSFD and to Australia with IMCRA);

- Otherwise, by default, the MEOW boundaries are used to subdivide the EEZ into Level 4 units.

High seas need a specific rationale for the definition of basic recording units. Under auspices of the Intergovernmental Oceanographic Commission (IOC) of UNESCO, the GOODS initiative presented (UNESCO 2009) a biogeographic classification for global open ocean and deep-sea areas compiled by an international expert group at a workshop held in Mexico City, Mexico, in January 2007. This was refined and formalised in a later paper by Watling et al. (2013), with 14 lower bathyal, 14 abyssal and 10 hadal provinces largely defined from abiotic characteristics and bathymetry. For the high seas, we aim to use as Level 4 units the deep-sea basins (below 3500 m depth) and the seafloor elevations (above 3500 m). Decisions are to be made on the treatment of overlapping claims resulting from unsettled borders. These (e.g. France/Spain in Bay of Biscay) could be appended to both adjacent geounits which therefore overlap, but this is not desirable because the total area of Level 4 units would be greater than that of the higher levels, whereas it should match exactly. The alternative, used in <marineregions.org> and retained herein, is to treat overlapping claims as a separate geounit.

### Genealogy of shapefiles

With the exceptions of Europe (MSFD subregions) and Australia (IMCRA regions), the default basis for coastal recording units will be version 12 of Maritime Boundaries (released October 2023) from Marine Regions (Flanders Marine Institute 2012). The shapefiles illustrating our concepts of the basic recording units are, therefore, obtained directly as the spatial objects of Maritime Boundaries v. 12 or by splitting of these according to the rationale here exposed. EEZs that are discontinuous will be split into as many discrete parts they comprise (e.g. Equatorial Guinea, Senegal, Malaysia). The regional datum used by MSFD shapefiles (ETRS89) and IMCRA (GDA94) differs from that (the worldwide WGS84) used in Maritime Boundaries v. 12, but this results in mismatches in the order of respectively 0.4 m and 2 m and will be ignored.

When the limits of MEOW are used for the subdivision of large EEZs, the original shapefiles of the MEOWs (hosted on The Nature Conservancy website <https://geospatial.tnc.org/maps/marine-ecoregions-of-the-world-meow> and also at <https://www.arcgis.com/sharing/rest/content/items/903c3ae05b264c00a3b5e58a4561b7e6/data>) are not used. There are several issues: (1) the inner limit is placed way inland and must be trimmed against a shapefile of the baseline; (2) the outer limit is not 200 NM, but an approximate contour and (3) terms of use include the commitment that “... you are using the data for non-commercial purposes, and *you don't alter them in any way*” (our italics), whereas we want to adjust our recording units to EEZs. Therefore, we will subdivide EEZs (from Marine Boundaries v. 12 at VLIZ) along the MEOW limits when appropriate, but will not use the MEOW shapefiles as such.

For the high seas, we aim to use as Level 4 units the deep-sea basins (below 3500 m depth) and the seafloor elevations (above 3500 m). Source shapefiles are those of the abyssal provinces of Watling et al. (2013), also hosted on the Marine Regions website (datum WGS84). In a first step, we intersected those shapefiles with the high seas polygons (thereby removing parts of the deep sea comprised in the EEZs, treated as “coastal”). We then partitioned the Level 2 “High Seas” polygon of each ocean in two complex polygons, one deeper than 3500 m and the other one shallower. Further subdivision of such polygons with many discrete tracks cannot be performed using a dividing line as was done for the EEZs; therefore, we created a set of templates, one for abyssal basins (running along the shallowest part of the dividing features) and one for bathyal seafloor elevations. These templates were intersected with the respective bathyal or abyssal shapefile.

### Syntax for geounits names and labels

Following section 3.3.3.1. of TDWG Standards Documentation Specification, the **term name** is a controlled value that represents the class, property or concept [here, concept] described by the term definition. In the case of WGSRPD and the Geoschemes proposed standards, those names correspond to the codes designating the units (one digit or letter for Level 1, two for Level 2, three for Level 3 and Level 4 names derived from Level 3 names with two more letters appended).

Taking into account that the marine proposal follows in the footsteps of WGSRPD, care must be taken to assign them a code with the same format and without duplicates between the land and sea proposals. In the WGSRPD, Level 1 units are identified by a one-digit number (1 to 9), Level 2 regions by a two-digit number (10 to 91), Level 3 units by a three-letter code (not related to the ISO-3166 code of the country) and Level 4 units by a five-letter code in the format XXX-YY where XXX is the Level 3 code and YY an identifier of the level 4 unit. Where Level 3 and Level 4 are identical (the case of 294 out of 614 Level 4 units in WGSRPD i.e. nearly half), this is denoted by a Level 4 code in the format XXX-OO (the double letter ‘O’). In WGSRPD, Level 4 parts of large countries (e.g. Russia) or not so large, but situated at crossroads (e.g. Egypt) may belong to different Level 3, Level 2 and Level 1 units and this also happens in the marine system (e.g. Egypt, Costa Rica).

Developing a set of similar codes for the marine part poses some challenges in order to avoid homonymy with terrestrial codes. Taking into account that the system is alphanumerical and that numbers in the Level 1 and Level 2 are already used up by the terrestrial units, we are compelled to use letters for marine Level 1 units (we propose R for Arctic, A for Atlantic, I for Indian, P for Pacific and S for Southern Ocean), then one digit appended to those letters (A1, A2 etc.) for the marine Level 2 units.

At Level 3, codes must all be distinct from the WGSRPD codes because the Level 2 and Level 1 units above in the hierarchy are different. We propose that the Level 3 code be composed of two letters (by default, the ISO 3166-2 code of the country if appropriate) and one digit, the presence of that digit unambiguously denoting a marine code (there are three letters in all WGSRPD codes). The digit is ‘1’ by default, but must differ if more than one unit is defined for the country or if the letters are already used in another Level 3 unit. At Level 4, the convention of using the suffix ‘-OO’ is conserved, otherwise diagnostic letters are used.

For high seas, it would have been tempting to use the Watling et al. (2013) provinces as codes for Level 3, but unfortunately this is not practicable because there are 14 provinces in the bathyal and 14 more in the abyssal, forcing some codes into four characters instead of three. Renaming them as B01 to B14 and A01 to A14 would help, but still this would not account for the fact that some Watling provinces straddle more than one ocean (Level 1 unit) and, therefore, must be subdivided. Taking into account these constraints, we propose Level 3 codes for the deep sea using the two characters of the Level 2 in which they are comprised and the third character a letter to discriminate the 28 provinces of Watling et al. (2013).

The local names as discussed above are the same as the **controlled value** strings (required for controlled vocabularies according to the TDWG Standards Documentation Specification). Admittedly, those codes, be they WGSRPD or marine, are not user-friendly. However, they are machine-friendly provided that they are unique and, therefore, they are needed as part of the standard. With respect to the human user, we have focused on the full names, which are treated as **labels**, with the intention to make them as easy as possible to identify a geographic area. Following section 3.3.3.1. of TDWG Standards Documentation Specification, the label is a word or short phrase that serves as a human-readable name for the term. These labels of coastal recording units will always start with the adjacent country or dependency name, for example, “France - Bay of Biscay”, not “French part of the Bay of Biscay”. We gathered that many users dislike adjectives (e.g. “Panamanian part of the Pacific Ocean”), which are sometimes awkward to pick in a pull-down menu.

Remote parts and/or dependent states (e.g. Martinique, Cocos Island) are named on their own in the case where they are identified by a separate ISO 3166 code, for example, “Portugal – Azores EEZ” (ISO code PT), not “Azores EEZ”, “Spain – Demarcación marina Canaria” (ISO 3166-2 code SP), not “Canaries EEZ”, but “Martinique EEZ” (ISO code MQ), not “France – Martinique EEZ”. The mention of “EEZ” in the proposed name of geounits is needed because, otherwise, there would be a confusion with the names of terrestrial Level 4 units of the same country; it is omitted if the part is explicitly named a sea area (e.g. “France – Bay of Biscay”, “Saudi Arabia – Red Sea”).

Therefore, the labels read as following:

**Country – EEZ** (for the complete EEZ of the country). Like in the Marine Regions website, “EEZ” is used instead of “Jurisdictional waters” even where no EEZ is formally declared (Mediterranean), for the sake of brevity.

**Country – IHOarea EEZ** or **Country – Ecoregion EEZ** when a large or complex EEZ has been subdivided according to IHO, MEOW or other scheme, for example, “Norway – Barentz Sea EEZ”; “Brazil – Rio Grande EEZ”.

**Country – MSFDsubzone** where applicable in Europe (we omit “EEZ” in this case).

**Australia – IMCRAprovince** where applicable around Australia (we also omit “EEZ”).

The naming of high seas features involves no country name but High Seas as such have the ISO 3166 alpha2 code QP (albeit not officially designated). Taking into account that the regionalisation of Watling et al. (2013) has been instrumental in the design of units, we use their sectors (BY1 to BY14 for areas less than 3500 m deep, see above and AB1 to AB14 for those deeper than 3500 m) as the first component of the label of high seas units, followed by the name of the undersea feature comprised in that unit, for example, “AB4 – Angola Basin”, “BY13 – Rio Grande Rise”, whenever possible using IHO’s “Gazetteer of Geographical Names of Undersea Features” <https://www.ngdc.noaa.gov/gazetteer/>.

### Hierarchy in geounits

In the system here proposed, hierarchy involves the same four strictly hierarchical levels as in WGSRPD. However, the implementation of this set of recording units in the Marine Regions website offers unlimited access to alternative hierarchies, because geounits in Marine Regions can be set with multiple parents. For Level 4 units, possible parents are: (1) the Level 3 unit of this proposal; (2) the nation to which it belongs and (3) the relevant general sea area. Level 4 units in the Marine Regions website can also be set as “parent” for smaller features (e.g. a Marine Protected Area), making that the data associated with such features will be retrieved in a query targeting that Level 4 unit or a more comprehensive one.

## Results

**Figure 1.**
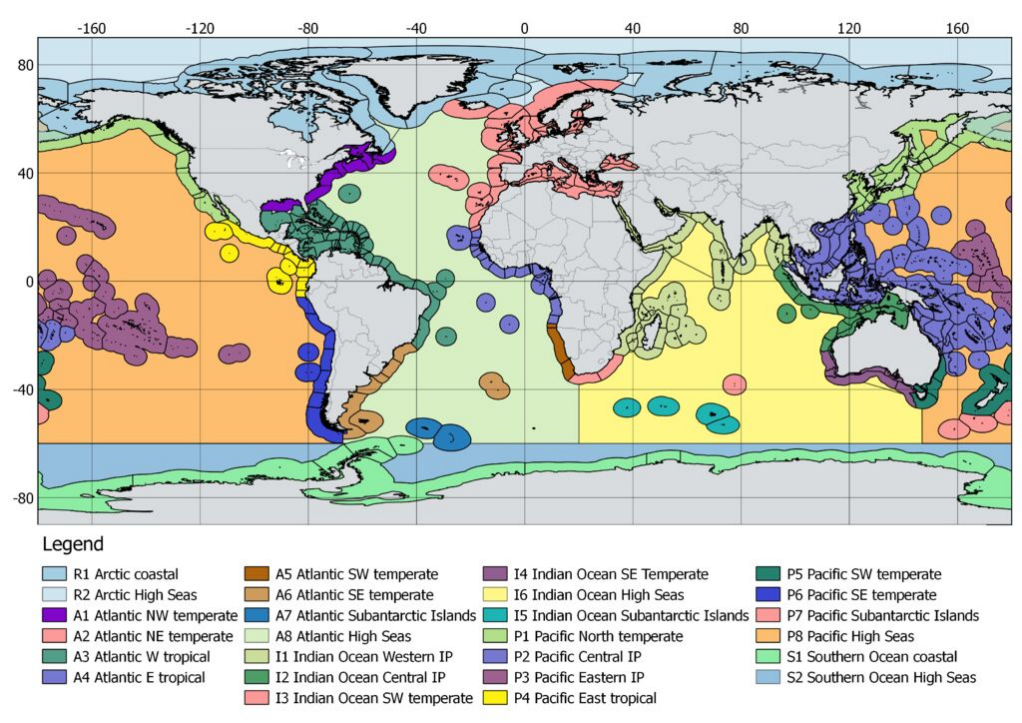
The proposed Level 2 units approximating the Realms of Spalding et al. (2007), showing also the boundaries of coastal Level 4 units. The coastal Level 2 units are adjusted to the nearest Level 4 unit and to a 200 NM seaward boundary.

Higher level units are here shown on Fig. 1, and the details at all levels are displayed on the GitHub repository, and in supplementary material to this article (Suppl. material 2).

### The proposal

The proposal for a marine scheme features five Level 1 units, 26 Level 2 units, 232 Level 3 and 536 Level 4 units (436 coastal and 100 in high seas), comparable to 9 Level 1, 52 Level 2, 372 Level 3 and 609 Level 4 in the current terrestrial scheme. This draft proposal is posted (TDWG Geoschemes task group 2023) on the GitHub repository with the purpose of gathering feedback. There, it is summarised on a series of maps, organised according to the Level 1 and Level 2 units in which they are comprised, followed by notes explaining some of the options taken. All the geounits are listed in the accompanying Excel spreadsheet (Suppl. material 3, also posted on the project's GitHub website) which, once amended and completed, will be the core proposal to TDWG once this public consultation is concluded.

Of the Level 4 basic recording units, 175 are coincident with the (potential) EEZ of a country or a dependent territory, 132 correspond to subdivisions of an EEZ following MEOW boundaries, 51 to subdivisions of an EEZ following IHO sea areas (i.e. the "Marine Region" placetype at VLIZ, not to be confused with the name of the website as a whole), 39 to Australian IMCRA areas, 18 to subdivisions according to European MSFD subareas and the 100 subdivisions of high seas are made according to the GOODS scheme.

### Higher level units

#### Level 1 units

In line with WGSRPD where Level 1 units are the continents, Level 1 units are the five oceans. Marginal seas (which are treated in the IHO scheme with the same rank as the major oceans) are appended to the major oceans following the boundaries set by NOAA (Eakins and Sharman (2020), see also our background document: TDWG Geoschemes Task Group (2022)) although other sources (e.g. Wikipedia) show slightly different groupings. Taking into account that the terrestrial Level 1 units used up single-digit codes 1 through 9, the codes for marine Level 1 units are here proposed as single-letter (A, I, P, R, S). Some Level 4 Basic Recording Units straddle two IHO ocean areas (e.g. a small part of Chilean EEZ extends into the Atlantic Ocean). In this case, we adjusted the limit of the Level 1 units to the nearest Level 4 limit, rather than splitting the Level 4 unit into two parts, which we deemed impractical.

#### Level 2 units

Level 2 units in the coastal area correspond broadly to the realms of Spalding et al. (2007) Marine Ecoregions of the World, defined therein as “very large regions (...) across which biotas are internally coherent at higher taxonomic levels, as a result of a shared and unique evolutionary history”. Their seaward limit is adjusted to the 200 NM and also adjusted to the nearest Level 4 unit (usually an EEZ boundary). Those realms (Temperate South America, Temperate Southern Africa, Temperate Australasia and Subantarctic Islands) which extend into more than one Level 1 oceans, are subdivided accordingly. Eastern and western parts of Temperate Northern Atlantic and Tropical Atlantic are also distinguished at Level 2. High Seas constitute an additional Level 2 unit in each Level 1 ocean. Codes for Level 2 regions will use a two-character format (the ocean’s letter and one digit).

#### Level 3 units

Level 3 units are identical to Level 4 in 125 cases and the other 107 Level 3 units account for the remaining 411 Level 4 units. The Baltic Sea, Black Sea, Lesser Antilles and the tropical part of Brazil are examples of Level 3 groupings.

#### Level 4: Basic recording units

Level 4 units never encompass the jurisdictional waters of more than one country or territory. For those countries with large and/or complex jurisdictional water, these are systematically split into as many units as there are Level 1 or Level 2 units involved (e.g. Costa Rica, Guatemala, albeit small, has one Pacific and one Caribbean unit; South Africa has one Atlantic and two Indian Ocean units).

In addition to this, some large EEZs (e.g. Brazil, Indonesia) are subdivided even if they are entirely contained in the same Level 2 unit by default. By default, partitions were made along the limits of ecoregions in the MEOW system of Spalding et al. (2007). Exceptions are places where there is a well-established local system of geounits with associated shapefiles: for Europe, the subregions defined for the Marine Strategy Framework Directive (MSFD; Jensen et al. (2017)); for Australia, the IMCRA regions (Commonwealth of Australia 2006) and an ad-hoc subdivision based on a published source was used for places where the MEOW ecoregions have been declared inadequate (e.g. Madagascar, Cooke et al. (2023)).

## Discussion

It must be emphasised here that the current project aims at establishing a **geographic** scheme to record distribution data, including those expressed as a text and is not primarily designed to provide a framework for biogeographic analyses. An immediate use for this set of basic recording units is its implementation in the “Distributions” factsheets of the World Register of Marine Species <www.marinespecies.org> (WoRMS). Actually, the present proposal was moved forward by WoRMS editors in a “Distributions Working Group” who recognised the lack of appropriate polygons to display distributions in the database and called for the selection of a coherent set of preferred geounits. WoRMS has stayed away from entering occurrence data based on unpublished datasets (leaving this endeavour to OBIS and GBIF) and, instead, relied on published sources and polygons within which at least one occurrence has been ascertained. The proposed set of geounits, compliant with political boundaries, as well as with current knowledge regarding biogeography, would enable the production of national or regional checklists of species for any country and any taxonomic group, once distribution factsheets are reasonably complete. In addition, the proposed recording units allow us to handle imprecise country-level records from the old literature. The completeness of mapping has so far been hampered by the lack of a consistent set of recording units, leading editors to use inappropriate one (too large, for example, “North West Atlantic” or countries displayed as land on the maps).

The proposal, as it stands, still needs polishing and this is why we strongly urge potential users to provide feedback and suggestions and to criticise or correct decisions we made in some parts of the World for which we do not have a first-hand knowledge. A few alterations were already made in this way, for example, the MEOW boundaries around Madagascar were disregarded because they do not reflect the uniqueness of the Madagascan deep South. The proposal, as it stands, is straightforward for many basic recording units, but we are not assertive for every detail and some points are still open for discussion. Following TDWG procedures, those points should be raised as “Issues” <https://github.com/tdwg/geoschemes/issues> on the proposal’s forum, which can be accessed by anyone using a GitHub account. We conclude this communication with warmly calling every interested scientist to give us feedback.

## Supplementary Material

A06FD2B8-F8E4-5912-89D1-1E22C665DD72 10.3897/BDJ.13.e157371.suppl1 Supplementary material 1 Document S1: Background for Groschemes marine proposal Data type Text and images Brief description Background document (an illustrated and expanded version of section 2. “State of the art: Existing sets of geounitsin the marine realm” of this article), also posted on the project’s GitHub repository: https://github.com/tdwg/geoschemes/blob/main/marine/background_v3_2025-07.md. File: oo_1373799.pdf https://binary.pensoft.net/file/1373799 Nicolas Bailly, Serge Gofas, Britt Lonneville

2EC9F99D-8358-53D5-B89B-5935689EEE4E 10.3897/BDJ.13.e157371.suppl2 Supplementary material 2 Document S2. Geoschemes marine proposal Data type Text and images Brief description Proposal document (an illustrated and expanded version of section 4. “Results: the proposal” of this article), also posted on the project’s GitHub repository: https://github.com/tdwg/geoschemes/blob/main/marine/proposal_v2_2024-12.md. File: oo_1373812.xls https://binary.pensoft.net/file/1373812 Nicolas Bailly, Serge Gofas, Britt Lonneville

5C6CA74B-0140-56AA-91C7-AD7D0C2C89D5 10.3897/BDJ.13.e157371.suppl3 Supplementary material 3 Table S1. Complete list of proposed geounits at levels 4 through 1 in Geoschemes marine Data type Data spreadsheet Brief description An Excel file describing all the proposed recording units in the TDWG Geoschemes marine proposal. File: oo_1358491.xls https://binary.pensoft.net/file/1358491 Nicolas Bailly, Serge Gofas, Britt Lonneville
