# Supplementary material for: A worldwide geographical scheme for recording the distribution of marine biota: proposal and call for feedback
Source: Biodivers Data J. 2025 Jul 31;13:e157371. doi: 10.3897/BDJ.13.e157371 (PMC12332496; doi:10.3897/BDJ.13.e157371)
Supplement: Supplementary material 1 — Document S1: Background for Groschemes marine proposal [file bdj-13-e157371-s001.pdf]

Supplementary material to article: **Bailly N., Gofas S., Lonneville B. "A worldwide geographical scheme for recording the distribution of marine biota: proposal and call for feedback"**
Background document (an illustrated version of section 2. “State of the art: Existing sets of geounits in the marine realm” of this article), also posted on the project’s GitHub repository <https://github.com/tdwg/geoschemes/blob/main/marine/background_v3_2025-07.md>

A worldwide geographical scheme for recording the distribution of marine biota: Background document

Introduction

More than thirty years ago, in the early stage of the Internet, the International Working Group on Taxonomic Databases (TDWG) recognized the need for an agreed system of geographical units at approximately “country” level and upwards for use in recording plant distributions. The deliberations over a period of three years of a committee involving taxonomists as well as applied botanists culminated in producing a standard (Hollis & Brummitt, 1992; Brummitt et al., 2001) which covers the whole world and has been widely accepted.

With an increasing wealth of data available in the marine realm, a similar standard is desirable for the sea. Geo-referenced data are accurately represented in such databases as the Ocean Biodiversity Information System, established 26 May 2000 (https://obis.org, see Grassle, 2000) and the Global Biodiversity Information Facility, established 2001 (https://www.gbif.org/, see) but there is also a need for pre-defined geographical units as a tool for (1) retrieval of point data and (2) handling the data formulated as the name of a country or region, with no more detail and no coordinates. Like for the terrestrial system, we believe that these should be politically bound in order to meet the needs of end-users.

With respect to continents, two peculiarities of the World Ocean make major differences. Firstly, it is a three dimensional realm where the water column can span thousands of metres and harbours a vertical succession of thoroughly different biotic communities. The vertical distribution of these communities is driven by factors such as light penetration and the characteristics of water masses. Also, the communities settled on the sea bottom (benthos) are radically different from those that inhabit the water column (plankton). On a map, those cannot be discriminated unless depth is added to geography.

The other major difference with the terrestrial realm is that only ca. 42% of the World ocean surface is under the jurisdiction of coastal states, whereas the remaining 58% are “high seas” where there are no political boundaries. Therefore, the rationale of using political boundaries must be complemented by decisions regarding the treatment of “high seas”.

The proposal is intended to parallel as much as possible the well established terrestrial scheme, about which Hollis & Brummitt (1992) wrote: “WGSRPD provides an agreed system of geographical units at approximately "country" level and upwards for use in recording plant distributions. It allows adopting organizations to compare and exchange data with each other without loss of information due to incompatible geographical boundaries. The system offered covers the whole world and identifies units at four levels, firstly continental, secondly regional (or subcontinental), thirdly at what may be called "Botanical Country" level (which may often ignore purely political considerations), and fourthly at a slightly lower level called "Basic Recording Units" where political integrity is fully recognised. (...) In many cases, where Botanical Countries have no complicating political factors, the units at Level-3 and Level-4 are identical. Very large countries, however, have been subdivided into more conveniently sized units according to constituent states or provinces.”

A wealth of marine (and also land) geographical units is stored, with associated descriptions and shapefiles, on the Marine Regions website https://www.marineregions.org/ at VLIZ, the Flanders Marine Institute. These units are classified according to “placetypes”, which are sets of geographical units established from the same source and/or with the same rationale. One of these “placetypes” is precisely the terrestrial TDWG scheme, and others with relevance to marine areas will be mentioned hereafter where appropriate.

In this paper, we will review existing sets of geounits defined in the marine realm and discuss their suitability, review current practice of selected end-users for the purpose of mapping distributions based on polygons, explain the rationale we intend to apply to the marine scheme and put forward a proposal for a suitable set marine geounits, with also four levels of hierarchy.

Rationale for the definition of units

The terrestrial scheme (second edition, 2001) has nine level-1, 52 level-2 , 372 level-3 and 609 level-4 units. In a hierarchical system, it is a requisite that the borders of more comprehensive levels coincide with those of included units, with no overlaps nor discrepancies. A similar hierarchy should be conceived for the marine counterpart with level 1 at the scale of the major Oceans. At the lowest level, a system designed to parallel the terrestrial system should always recognize the marine jurisdictional waters of one country, however small, as a unit, but should split those of large and/or complex countries into as many units as found desirable (e.g. Costa Rica, Guatemala, albeit small, should have one Pacific and one Caribbean unit; South Africa, Brazil or China which have continuous but very extensive coastlines spanning faunal breaks should be split). Taking inspiration from the terrestrial system, level 3 may accommodate groups of level 4 units which share political, biogeographical and/or geographical features (e.g. the Caribbean leeward islands, each of which would be a level 4 unit), but may be coincident with level 4 units when no obvious grouping is suggested. The development of level 2 units is not straightforward and will be discussed hereafter.

For the marine coastal Level 4 units, “coastal” is understood as the 200 nm EEZ or potential EEZ, regardless of the extension of the physical continental shelf, regardless of non-claimed EEZs (e.g. Greece) and not including claims to an “extended continental shelf” in the sense of UNCLOS. A decision must be made for overlapping claims (e.g. France/Spain in Golfe du Lion, Spain/Portugal around Selvagens); one possibility, presumably best for end-users, is to let them overlap.

No attempt will be made to represent separately any particular habitats (e.g. the rocky shore, coastal marshes, the bathyal level, the water column). Admittedly, it can be found shocking to represent the distribution of a limpet which is strictly coastal as a 200 miles fringe, however, we concluded that the advantages of using distinct sets of recording units for representing the distribution of organisms living in different compartments (e.g. intertidal vs. subtidal, pelagic vs. benthic) are by far outweighed by the advantage of having a universal scheme. Our final consideration is that if a species is reported in the “Basic Recording Unit” it is understood to occur “somewhere” therein, not “everywhere” therein.

Existing sets of geounits in the marine realm

In this section, we discuss some of the existing schemes for subdivision of the marine realm. and discuss their suitability as potential recording units. Most of these are also listed by Sayre et al. (2017: Table 1) and Zhao et al. (2020: Table 1), Some schemes are exclusively political (e.g. EEZ, MSFD), geographical (e.g. IHO, GOODS), others have a more or less explicitly biogeographical background.

IHO

The International Hydrographic Organization has published a detailed geographical scheme for subdivision of the World Ocean (IHO, 1953) into named sea areas, for which shapefiles are stored on the MarineRegions website (Figure 1).

The largest IHO polygons could be candidate geounits for Level 1 of a universal marine scheme, if they encompassed the entire oceans, but this is not the case as many parts of oceans and seas are named as separate geounits of the same level (e.g. Gulf of Guinea and Bay of Biscay are not part of the North Atlantic Ocean, but adjacent units of same rank). Therefore, the IHO scheme could be used only if adapted by merging all the smaller adjacent polygons to the main Oceans. The Marine Regions website has a placetype “General Sea Area” where polygons are first level child geounits of “World Oceans”, but there also many local units are unbalanced as for their place in the hierarchy.

There is also an initiative called SeaVoX
https://www.bodc.ac.uk/resources/vocabularies/seavox/, largely similar to the IHO scheme but with North Atlantic, South Atlantic and South Pacific further divided into East and West

parts. There also, Level 1 is utterly unbalanced with (mostly the same) adjacent area being on the same level, not part of the major oceans.

All this considered, the groupings of marginal seas as used by NOAA (Eakins & Sharman, 2010) could be used (Figure 2).

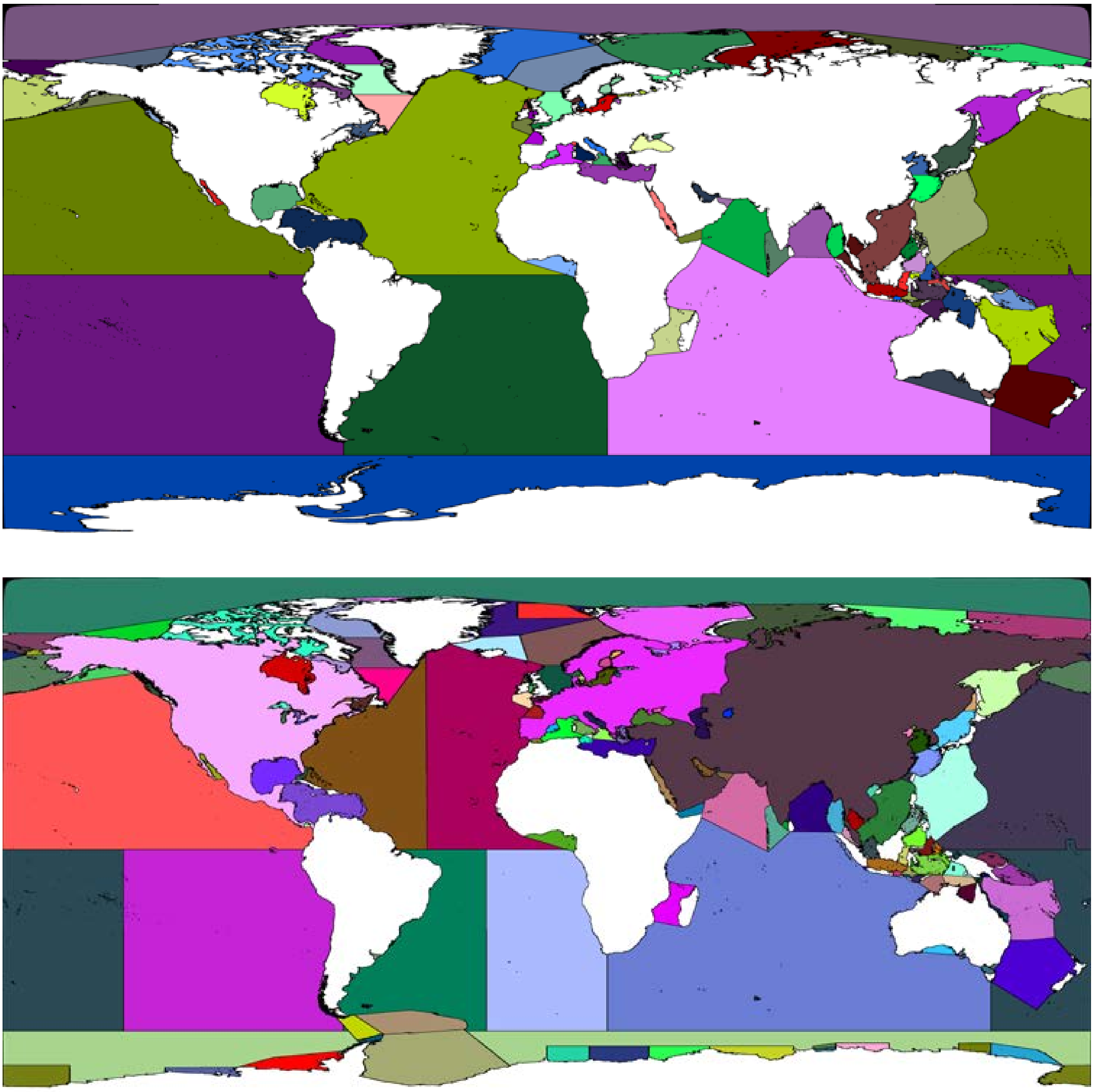

Figure 1. The IHO polygons (this representation downloaded from GITHUB forum gbif/pipelines#612, accessed 2022-05-26) (below, the SeaVox areas for comparison)

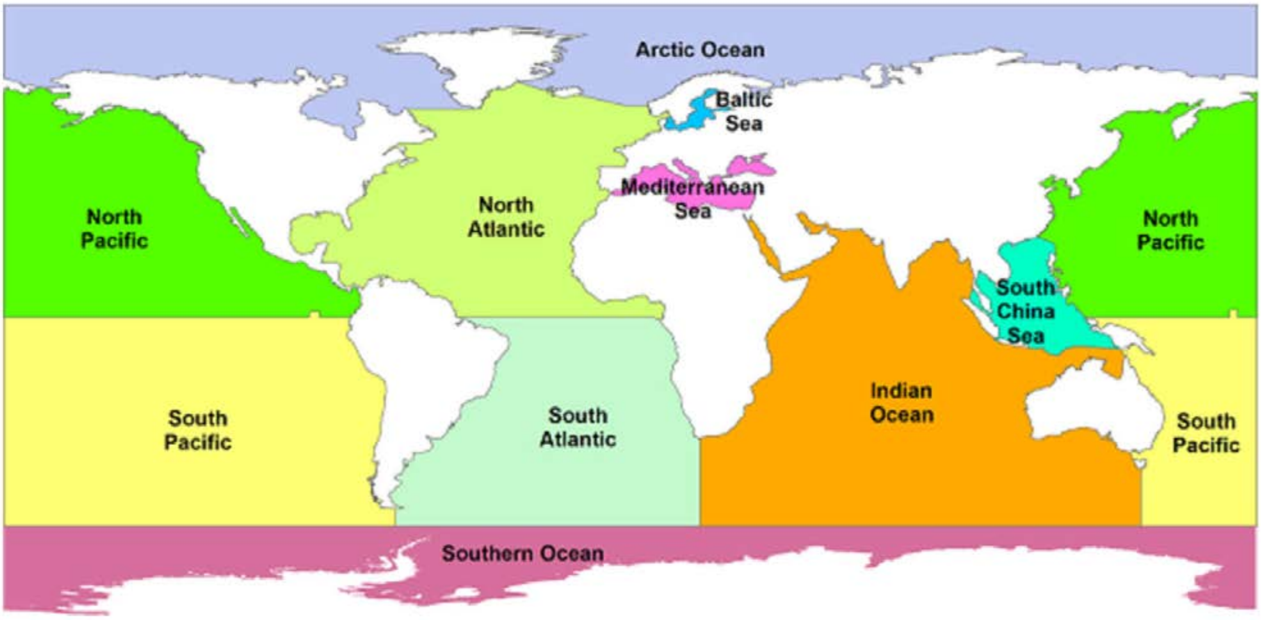

Figure 2. The major oceans and sea areas as grouped from the IHO scheme by NOAA; South China Sea could be further grouped with the Pacific Ocean and the Baltic, Mediterranean and Black Seas with the Atlantic

Exclusive Economic Zones (EEZs) and the "Marine Region" placetype at VLIZ

The EEZs are jurisdictional waters declared by coastal states under the provisions of the United Nations Convention for the Law of the Sea (UNCLOS). For those states which have not declared so far an EEZ, the UNCLOS considers the concept of a "continental shelf" (not constrained to the physiographic continental shelf) which is granted with no need for a declaration and comprises the sea-bottom to the same extent of 200 nautical miles. In the Marine Regions website, "EEZ" is taken in the broad sense of a potential EEZ from shore to a distance of 200 NM, and a complete set of shapefiles is publicly available at <https://www.marineregions.org/downloads.php> (latest version 12, October 2023).

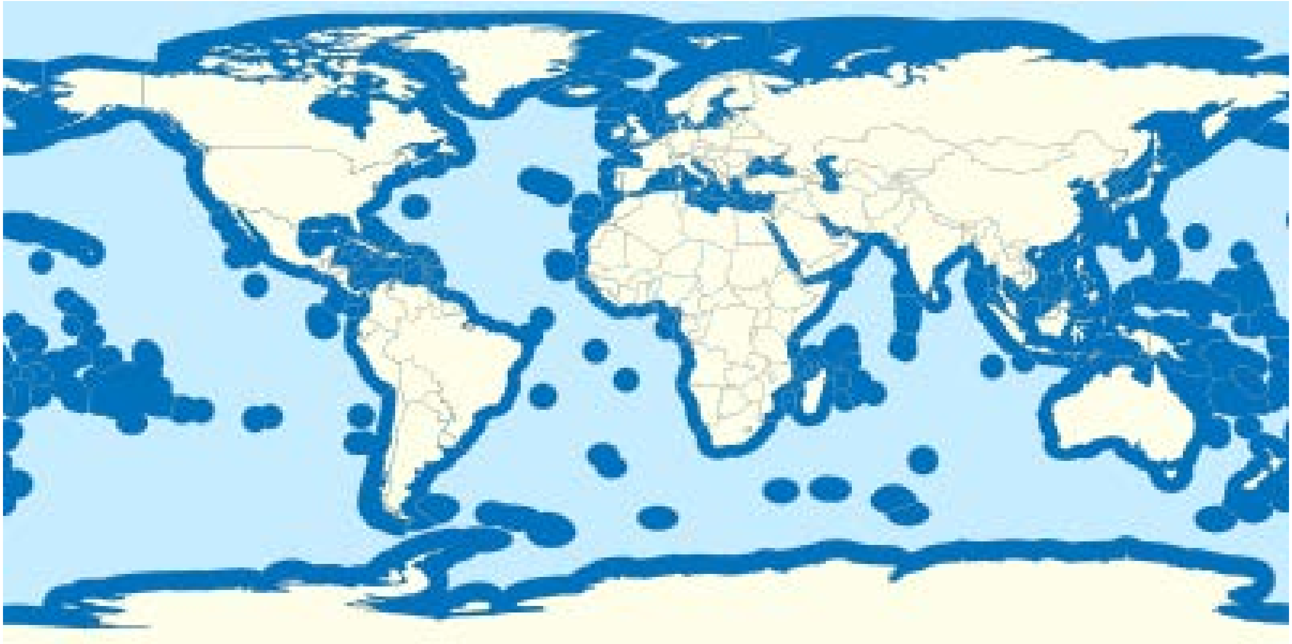

Figure 3. The world EEZs

For many small or medium-sized coastal countries and territories, the corresponding EEZ provides a suitable polygon for the basic recording unit at level 4. The EEZs of larger countries (e.g. The United States, South Africa, Indonesia…) and/or with a complex maritime facade (e.g. Spain, Egypt, Costa Rica…) must be split.

The Marine Regions website at VLIZ <www.marineregions.org/> has developed a set of geounits with placename “Marine Regions” (not to be confused with the name of the website as a whole) which is the intersection of EEZs with the IHO sea areas. These are straightforward for some countries with two maritime facades (e.g. Costa Rica) but there are issues which preclude using universally this set of geounits when we need to subdivide EEZs

(1) as a consequence of IHO severing adjacent sea areas from larger oceans or seas, some EEZ/IHO intersections result in awkward representations (e.g. “Egyptian part of the Red Sea” in Marine Regions misses Gulf of Suez, and Gulf of Aqaba, deemed to be different bodies of equal rank to “Red Sea” in the IHO Scheme).

(2) in other cases, the intersection of EEZ with major Oceans in the IHO scheme are too large (e.g. United States part of the Atlantic Ocean, spanning from Maine to Florida), therefore inadequate as basic recording units.

Under certain conditions, countries apply for an “Extended Continental Shelf” beyond the 200 miles but no further from the baseline than either 350 miles, or 100 miles beyond the 2500 m isobath (whichever is more favourable; see Wu et al. 2013); this field is currently in a state of flux and is not taken into account in the present project.

Marine Ecoregions of the World (MEOW) (Spalding et al., 2007)

Supported by the publication by Spalding et al. (2007), this scheme aims to a biogeographic classification of the world's coastal and continental shelf waters following a nested hierarchy of nested system of 12 realms, 62 provinces, and 232 ecoregions (with an average surface of 685.345 km^2). Three principles were followed for this scheme: that it should have a strong biogeographic basis, offer practical utility, and minimize discrepancies with existing systems. At first glance, MEOWs provide units with a size largely suitable as “Basic Recording Units” (level 4) for the coastal regions. Their main drawback is that they ignore country boundaries and must therefore be adapted (intersecting a MEOW with an EEZ) if a country-based system is wanted.

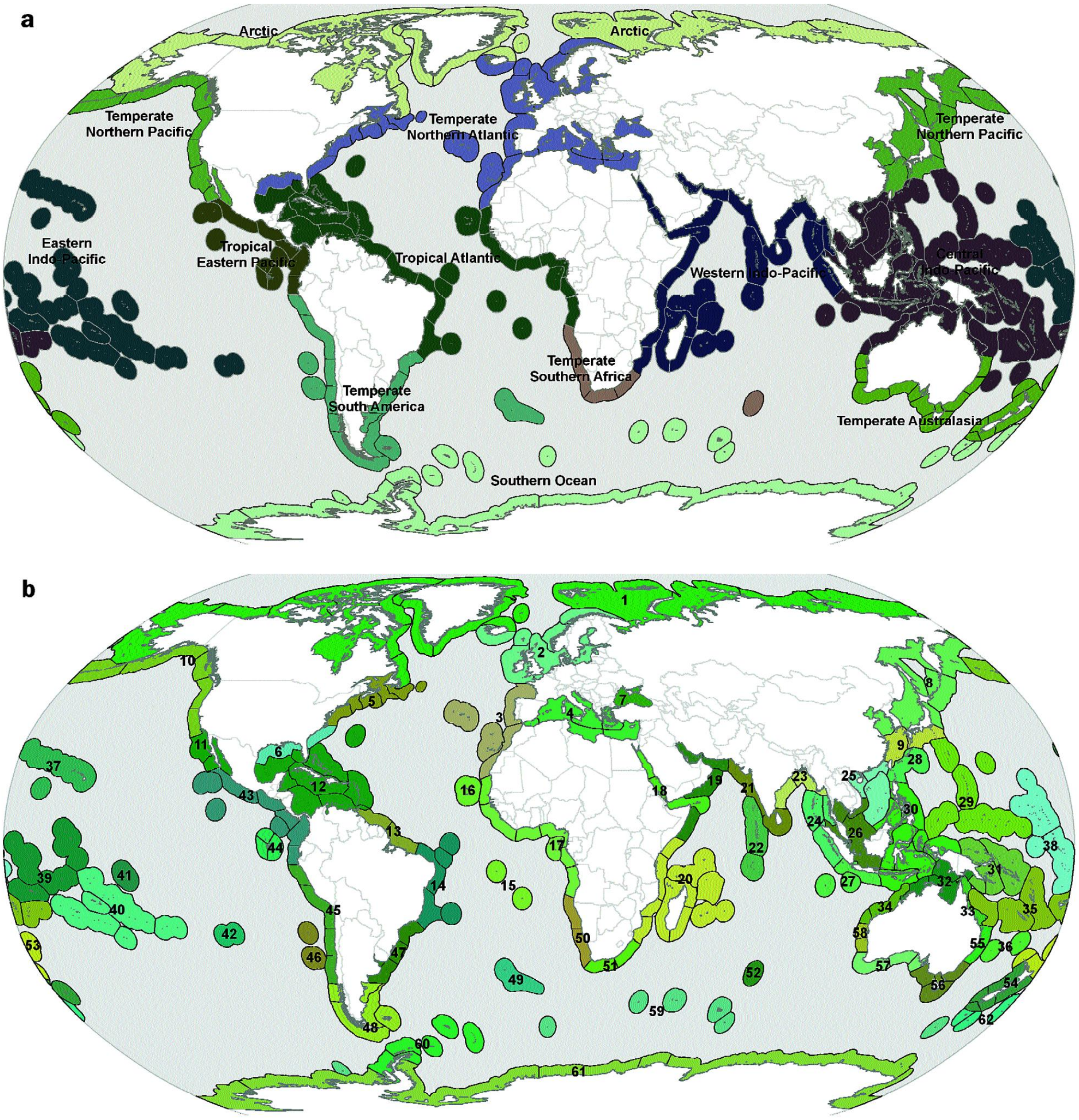

Figure 4. Final biogeographic framework of the MEOW system. (a) Biogeographic realms with ecoregion boundaries outlined. (b) Provinces with ecoregions outlined (reproduced from Spalding et al. 2007, where numbers are explained)

Original shapefiles of the MEOWs are hosted on The Nature Conservancy website https://geospatial.tnc.org/maps/marine-ecoregions-of-the-world-meow. The outer boundaries are close to, but not coincident with the 200 nautical miles of the outer boundary of EEZs. The inland boundaries of the shapefiles extend another 200 miles inland from the coastline - a convention to ensure inclusion of any estuarine/lagoonal systems. The Marine Regions website hosts shapefiles of the MEOW intersected with the coastline so as to include only the marine part.

Regional schemes

Coastal countries belonging to the European Union have commitments towards achieving a “Good Environmental Status” of their jurisdictional waters, as detailed in the Marine Strategies Framework Directive. This generates a need for environmental monitoring and elaboration of a suite of 11 “descriptors”, and the MSFD regions were defined as a framework for reporting ((Jensen, Panagiotidis & Reker, 2017). Countries with a composite EEZ spanning two or more of these regions (Sweden, Germany, France, Spain, Italy, Greece) have divided their jurisdictional waters into subregions according to this scheme. These administrative boundaries used in the context of the E.U. Marine Strategies Framework Directive are highly suitable as “Basic Recording Units” and will be used in this proposal. For Spain, these polygons have been already used for the establishment of an official reference list of all marine organisms present in the country, issued in 2017 (but unfortunately withdrawn in 2020).

Another well-established regional scheme is Australia’s “Integrated Marine and Coastal Regionalisation of Australia” (IMCRA 4.0). There are only seven (the states) Level 4 units (the states) in the terrestrial WGSRPD scheme but the marine realm is complex. No less than 18 MEOW are defined in Australian waters (Spalding et al., 2007). An alternative scheme is provided with the “Integrated Marine and Coastal Regionalisation of Australia” (IMCRA 4.0)and 41 regions in the marine IMCRA 4.0 <https://www.environment.gov.au/system/files/resources/2660e2d2-7623-459d-bcab-1110265d2c86/files/map1-pb.pdf>, which departs from an other EEZ-based schemes by considering separately the shallow coastal regions and the remainder of the EEZ. However, it has the advantage of being a coherent system whose outer boundaries are exactly coincident with Australian EEZ. The designation of six marine regions (North, Coral Sea, Temperate East, South-east, South-west and North-west) defined in support of the Environment Protection and Biodiversity Conservation Act was supposedly informed (Evans et al. 2016) by the provincial bioregions identified as part of the IMCRA 4.0, but the boundaries are not coincident, the “transition zones” recognized in the IMCRA scheme are arbitrarily split and the scheme even departs from traditional Australian marine biogeographical regions (Poore & O’Hara 2007). The important point is to respect the contrast between temperate and tropical parts.

IMCRA regions are used for display in the Australian Faunal Directory <https://biodiversity.org.au/afd/home>, whereas Atlas of Living Australia (Belbin et al. 2021,

<https://www.ala.org.au/>) uses only point data for the display on species factsheets but uses the IMCRA regions as bounding box for search within an area <http://regions.ala.org.au/#rt=States+and+territories>
A further reason for adopting them IMCRA regions as Level 4 units is that, despite their number, their extension is similar to that of Level 4 units in many other parts of the World (average surface ca. 244,000 km2).

IUCN Bioregions

A two-level scheme was established by Kelleher et al. (1995) with 18 bioregions and 136 "Biogeographic zones" for the purpose of assessing the geographical representativeness of MPAS. It is noteworthy that IUCN itself does not use these geounits in the maps representing the geographic range of the species assessed in the Red List, but instead uses a customized representation of the distribution of coastal species on a width of less than half the extension of the EEZ. Some articles on biogeographical topics (e.g. Hewitt et al., 2011, Swart et al. 2018, Castro et al. 2020) nevertheless used these IUCN Biogeographic zones. They are also used for the display of distributions in the invasive species database NEMESIS https://invasions.si.edu/nemesis/ (Fofonoff et al. 2018) although this is not explicitly stated. Drawbacks are the same as for the MEOW realms and ecoregions, with the aggravating circumstance that they are far less well-known.

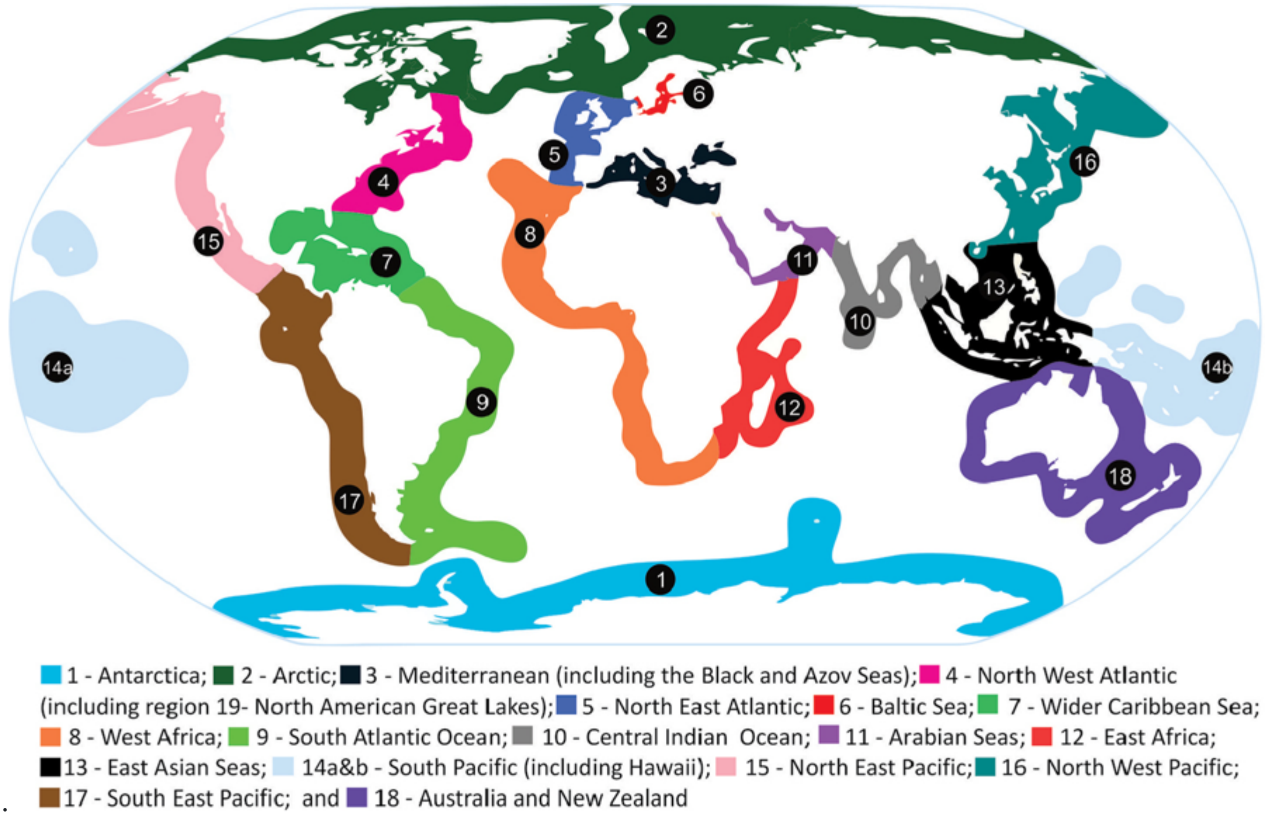

Figure 5. Map illustrating the 18 IUCN bioregions defined by Kelleher et al. (1995a, b, c, d), figure modified from Hewitt et al. in Swart et al. 2018, NeoBiota 39: 79–102, doi: 10.3897/neobiota.39.22002

Bioregions2020

There is an online-only initiative named “Bioregions2020” https://www.oneearth.org/bioregions-2020/, promoted by the NGO One Earth, which claims working towards a unified (terrestrial/marine) scheme. One major benefit of this bioregional framework, according to its authors, is that it allows for the integration of all three types of ecoregions – terrestrial, freshwater, and marine -- into a cohesive system. For the marine part, it draws mostly from MEOW so that we do not see any benefit with respect to the well-known scheme of Spalding et al. (2007). Anyway, we could not trace any supporting scientific publication regarding this scheme.

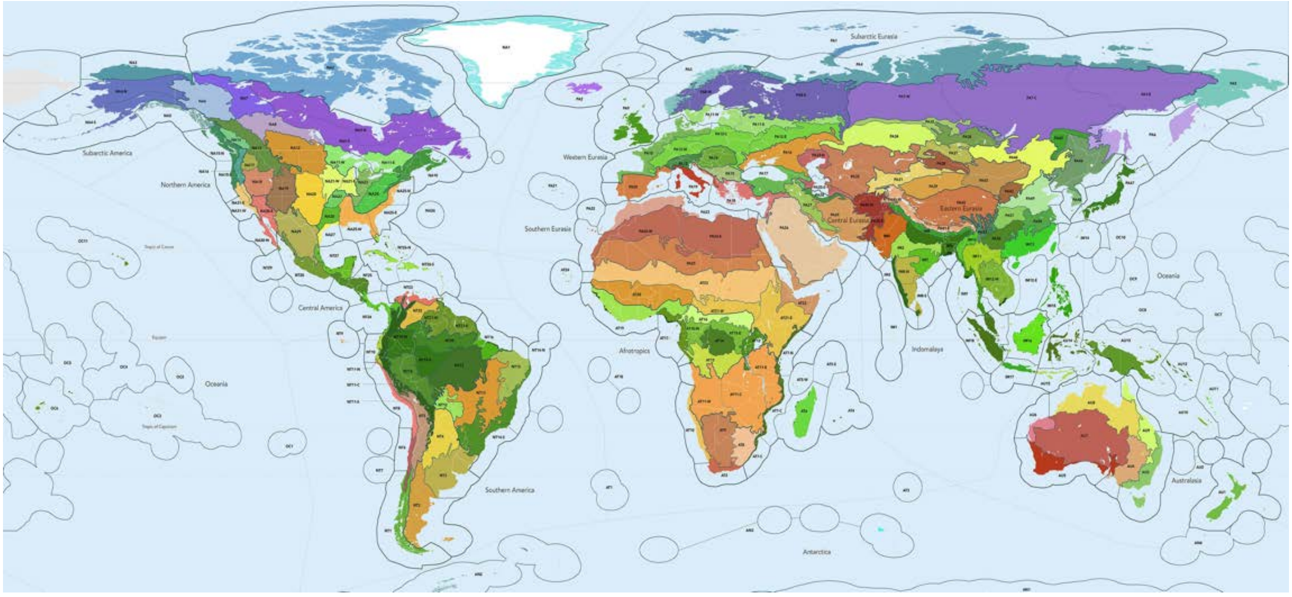

Figure 6. A map of the Earth showing the 185 discrete bioregions of Bioregions2020. Credit: Karl Burkart, One Earth

The 185 bioregions in the OneEarth system are organized by the world’s major biogeographical realms, the broadest division of the Earth's land surface within which groupings of organisms share a common evolutionary history -- Nearctic, Neotropic, Palearctic, Afrotropic, Indomalayan, Australasian, Oceanian, and Antarctic. Bioregions cannot cross a realm line, so each realm contains a finite number of bioregions, which can be grouped into subrealms.

This is essentially a terrestrial minded project, therefore major oceans are split according to essentially terrestrial boundaries, and opposite marine facades of major continents are lumped in the same realm whereas e.g. West and East Africa belong to different first level subdivisions in the sea.

Longhurst pelagic provinces

The development of remote sensing technology makes it possible to evaluate the concentration of chlorophyll based on the solar radiation reflected by the oceans, this is "Ocean-Colour Radiometry" (OCR) e.g. the Sea-viewing Wide Field-of-view sensor (SeaWiFS) project (Hooker & McClain, 2000). The relationship between chlorophyll and biomass is not fixed but depends on the taxonomic composition of the plankton components and, therefore, on the region: Hence the need for a partition of the world ocean into areas where this relationship is stable.

In this perspective, Longhurst (1995) recognized four main biomes at a first level: Polar, western, trade wind and coastal biome, recognizable in all ocean basins. At a second level, each basin is divided into provinces that provide a model for data analysis, for example assigning the necessary parameters to calculate biomass from OCR data. With their very specific focus on the open water, Longhurst provinces are not suitable as recording units on a global scale and will not be considered further.

Large Marine Ecosystems

The LME approach is aimed to improve the management of ecosystem goods and services. *Large Marine Ecosystems* (Sherman, 1991) are adjacent to continents in coastal waters where primary productivity is generally higher than in the open ocean. The physical extent of the LME and its boundaries are based on four linked ecological criteria, rather than political or economic ones. These are: (i) bathymetry, (ii) hydrography, (iii) productivity (iv) trophic relationships. Based on these 4 ecological criteria, 64 different LMEs have been delineated around the Atlantic, Pacific, and Indian coastal margins.

FAO Major Fishing Areas for Statistical Purposes

These are arbitrary areas (FAO Coordinating Working Party on Fishery Statistics 2025), the boundaries of which were determined in consultation with international fishery agencies and which are designed to coincide, as far as possible, with the areas of competence of fishery commissions. A total of 19 major marine fishing areas have been internationally established to date, covering the waters of the Atlantic, Indian, Pacific and Southern Oceans with their adjacent seas, being used since the 1950s for reporting the global fisheries landings.

These areas span both EEZs and High Sea and their limits are not systematically coincident with political borders so that, both because of this and because of their coarse resolution, we do not find them appropriate for a more fine-grained recording of distributions.

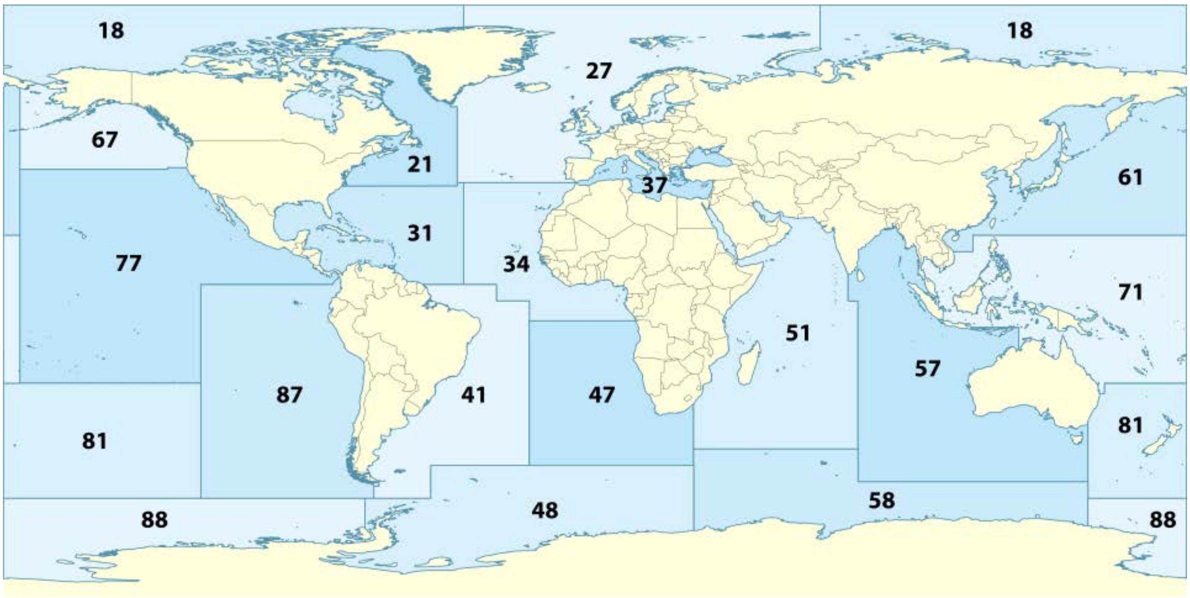

Figure 7. The FAO Major Fishing Areas for Statistical Purposes

Global biogeographic schemes

Global regionalizations of coastal waters were pioneered by Ekman (1935, 1953). They are based on the analysis of actual distributions of marine species, usually those belonging to well-known groups such as fishes, which reliably cover the entire marine realm. Briggs (1974) and Briggs & Bowen (2012) ranked regions at the highest hierarchical level, and recognized provinces (mapped in Toonen et al., 2016) based on an arbitrary threshold of 10% endemic species (of fish, in their data).

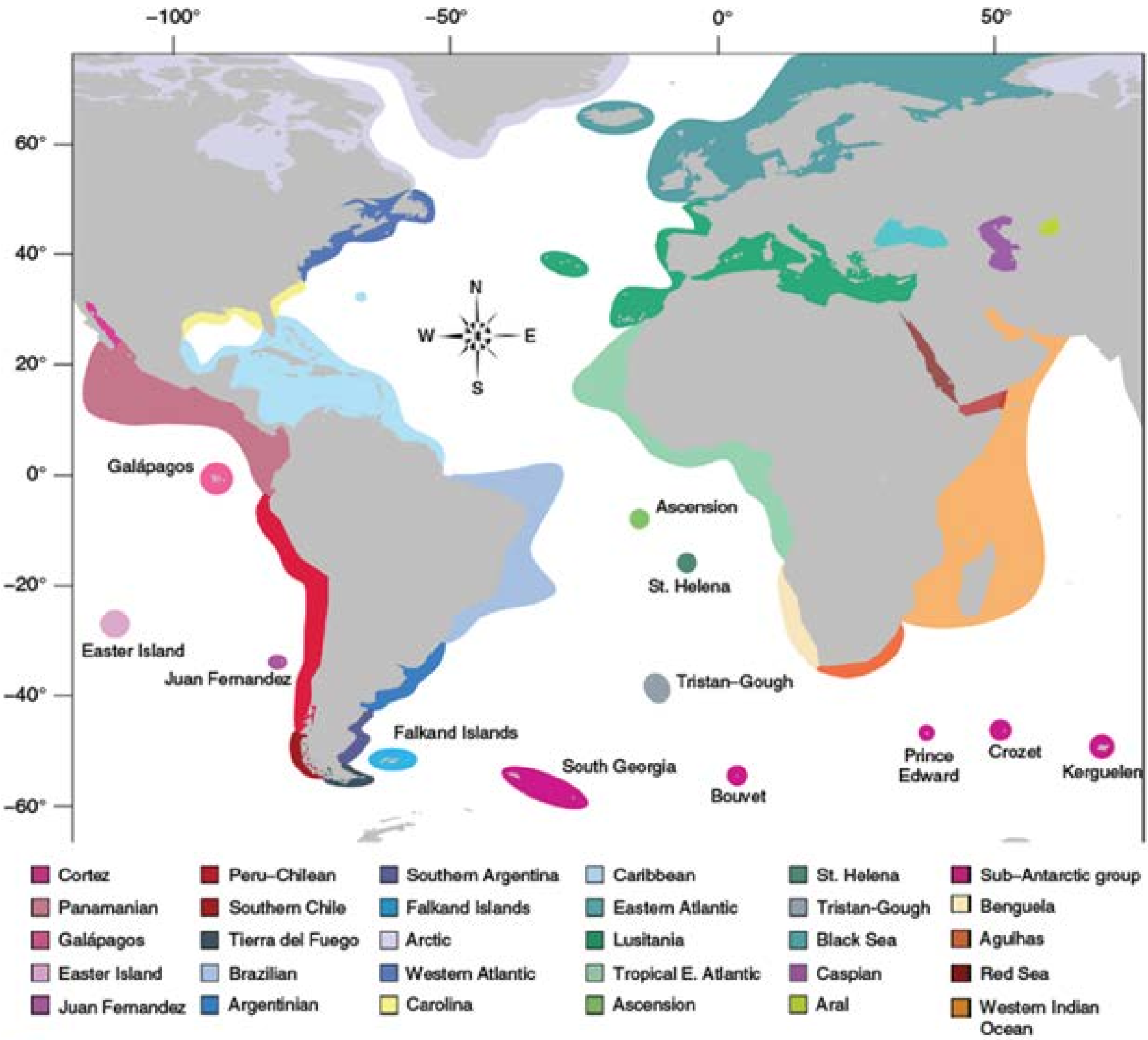

Figure 3 Map of the Atlantic and East Pacific Provinces. Each province is identified by a distinct color as shown in the key below the map. In general, the key lists provinces from West to East and from North to South. The Antarctic Province is outside the range of the map and is not shown here.

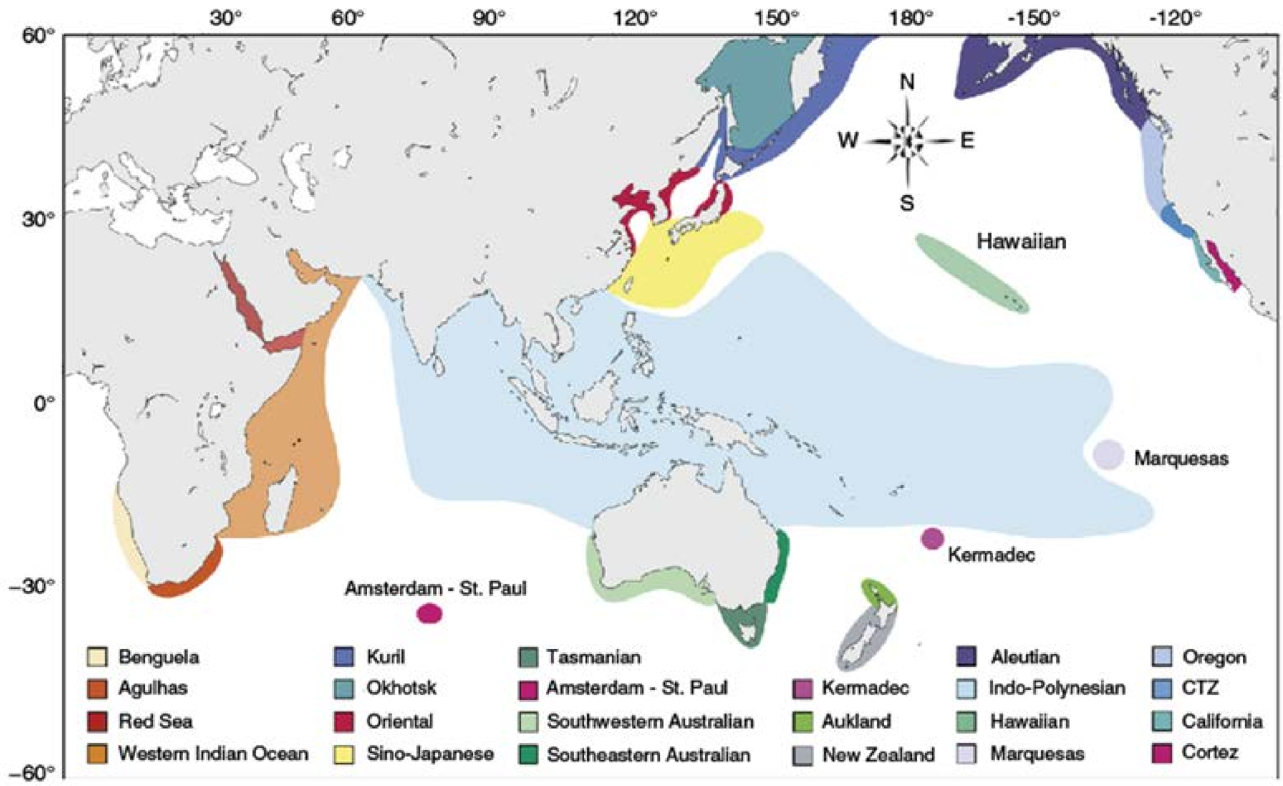

Figure 2 Map of the Indo-West Pacific Provinces. Each province is identified by a distinct color as shown in the key below the map. In general, the key lists provinces from West to East and from North to South. One province from the subantarctic region (Macquarie) and one of the New Zealand–Australian region provinces (Antipodes) are outside of the range of this map and are not shown.

Figure 8. A global regionalization of coastal waters (reproduced from Toonen et al. 2016)

The Marine bioregions designed by Watling & Gerken (1999) in the context of OBIS https://web.archive.org/web/20110611033825/https://marine.rutgers.edu/OBIS/biogeo/watling.htmare no longer visible on the Internet but follow basically the views of Briggs (1974) which are sensible

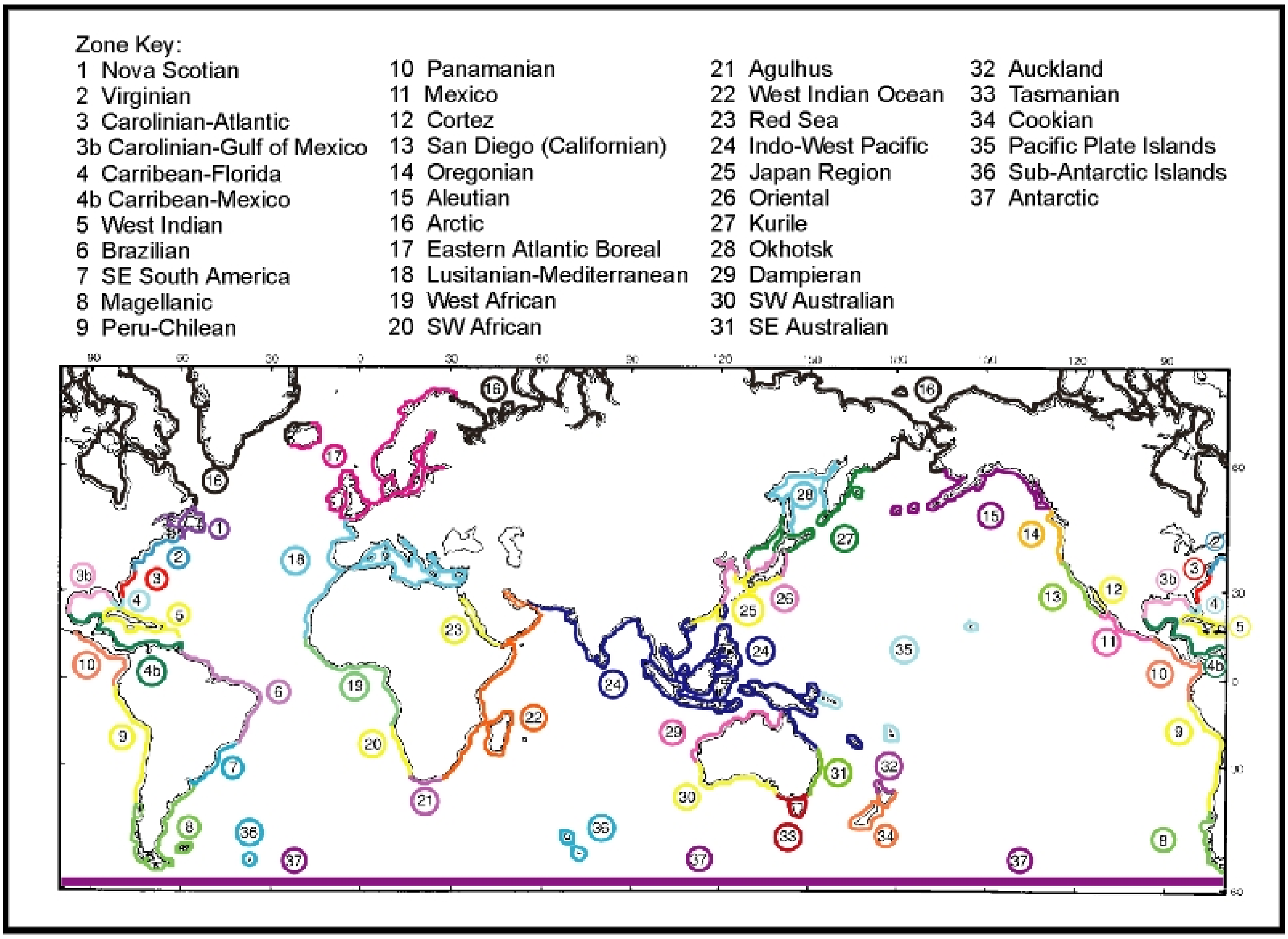

Figure 9. The Marine bioregions designed by Watling & Gerken (1999) as captured on the Internet Archive June 11, 2011

Data-driven regionalization schemes

With the increasing amount of Geo-referenced data contained in the Ocean Biodiversity Information System (OBIS) and the Global Biodiversity Information Facility (GBIF), attempts are being made for the categorization of biogeographic realms based on the statistical analysis of the distribution of occurrence data worldwide, without any a-priori grouping (e.g. Costello et al. 2017, Neubauer et al. 2025). However such regionalization schemes have limitations (discussed at length in Neubauer et al. 2025) derived mostly from the utterly unequal coverage of taxa and areas in the occurrence databases (Kennedy & Rotjan 2023). Costello et al. (2017), based on the distribution of 65,000 species of marine animals and plants, both benthic and pelagic, found 30 distinct marine realms but some of them span both high seas and coastal areas, and none are coincident with the 200NM used in the political subdivision. Therefore, even if we acknowledge the potential of such analyses, we find premature their integration in an essentially pragmatic set of polygons designed for the purpose of recording and retrieving species distributions.

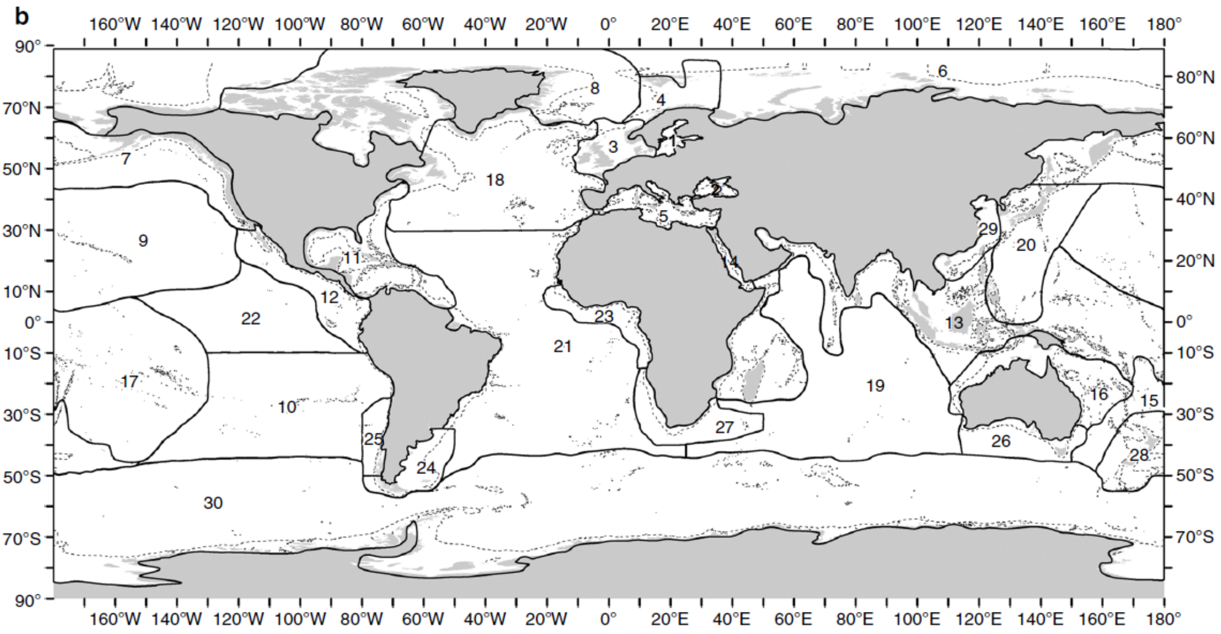

Figure 10. The biogeographic realms 1–30 of Costello et al. (2017). Realm boundary smoothing included following the Southern Ocean 10°C annual average sea surface temperature sub-Antarctic Front. Dashed line represents the approximate 1000m depth contour (reproduced from Costello et al. 2017 under CC BY-NC-ND license

GOODS and Watling et al. regions for the deep sea

As mentioned earlier, 58% of the marine realm does not belong to “jurisdictional waters” and cannot be treated with the same rationale as the coastal areas. Under auspices of the Intergovernmental Oceanographic Commission (IOC) of UNESCO, the GOODS initiative presented (UNESCO, 2009) a biogeographic classification for global open ocean and deep sea areas compiled by an international expert group at a workshop held in Mexico City, Mexico, in January 2007. This was refined and formalized in a later paper by Watling et al. (2013), with of 14 lower bathyal, 14 abyssal and 10 hadal provinces. The shapefiles for the GOODS system are available on the Marine Regions website.

Watling et al. (2013) noted that most of the upper bathyal (300-800 m) is within national EEZs, and considered separately the lower bathyal (plotted as 801–3500 m), the abyssal (3500–6500 m) which comprises 65.4% of the world’s seafloor, and the hadal (ultra-abyssal) areas (>6500 m). A major issue is raised because the GOODS system uses physiographic boundaries, essentially the isobaths, and largely overlaps with the 200-miles defined coastal regions. Therefore, the GOODS geounits, in order to fit the general TDWG scheme, should be intersected with the EEZs so as to prune the overlapping parts.

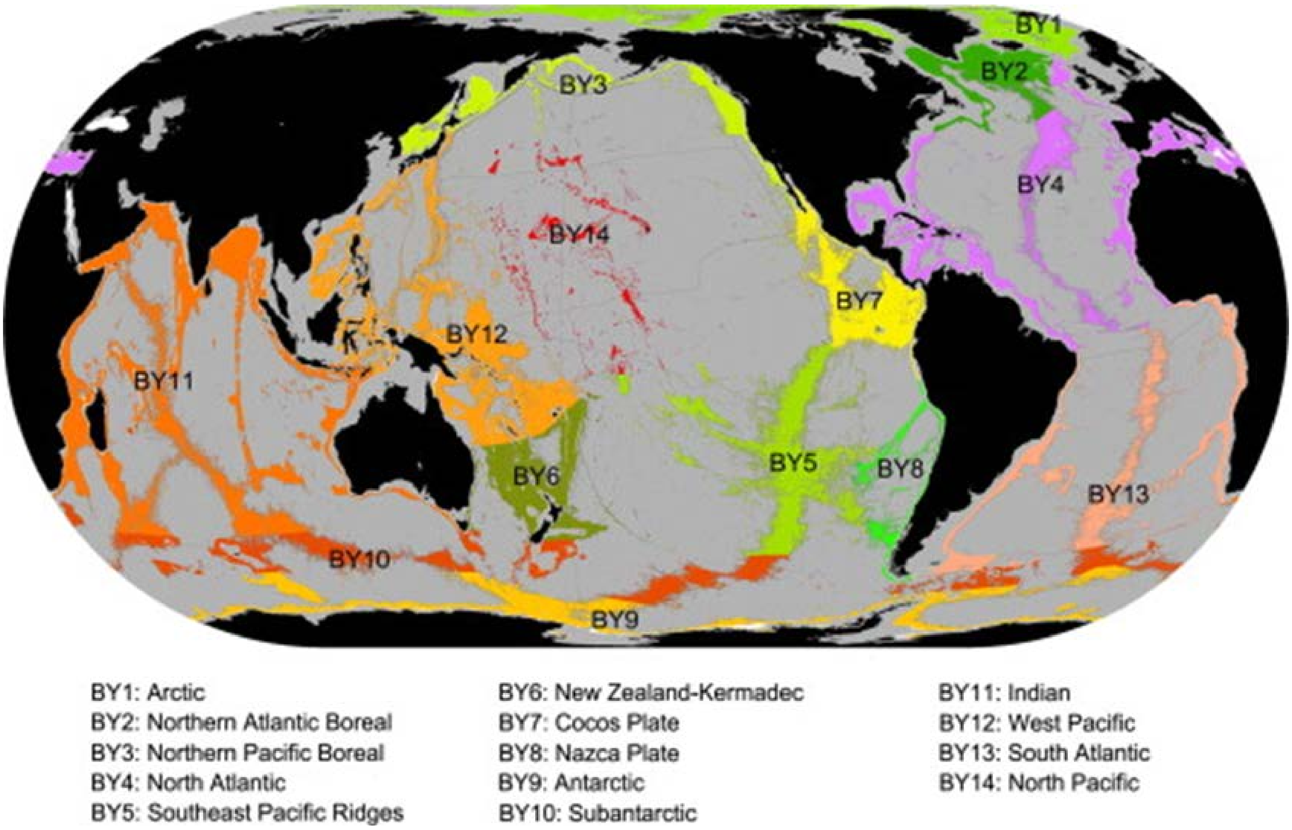

Figure 11. Lower bathyal provinces (reproduced from Watling et al. 2013)

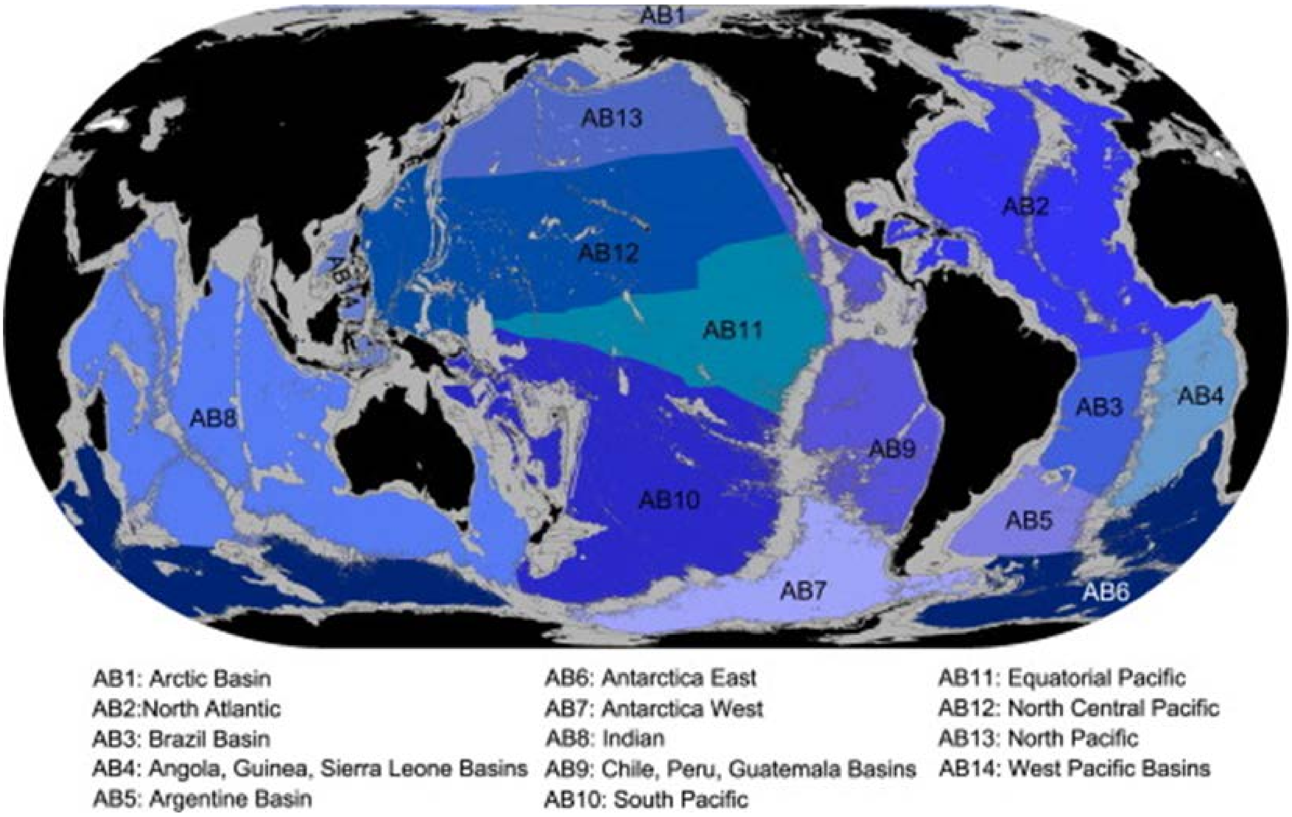

Figure 12. Abyssal provinces (reproduced from Watling et al. 2013)

From a pragmatic point of view, if major oceans are taken as Level 1 in the proposed scheme, “High Seas” of each ocean can be Level 2 geounits, and the Watling et al. provinces (constrained to the high seas part) used at level 3 or 4.

A sensible way to subdivide the large abyssal provinces of Watling et al. (2013) could be using physiographic basins, largely used in some geological (e.g. Emery et al. 1975) and taxonomic works (e.g. Allen, 2008), in which cases the Watling provinces may be used at level 3 and the individual seafloor features and basins at level 4.

A geomorphic features map (GSFM) of the global ocean was elaborated by Harris et al. (2014) but this research focuses the differences between active and passive margins, not on regionalization as such. Statistics regarding undersea features are presented for each of the major oceans, but features are not named nor considered individually, therefore not bringing any helpful element for defining recording units at Level 4.

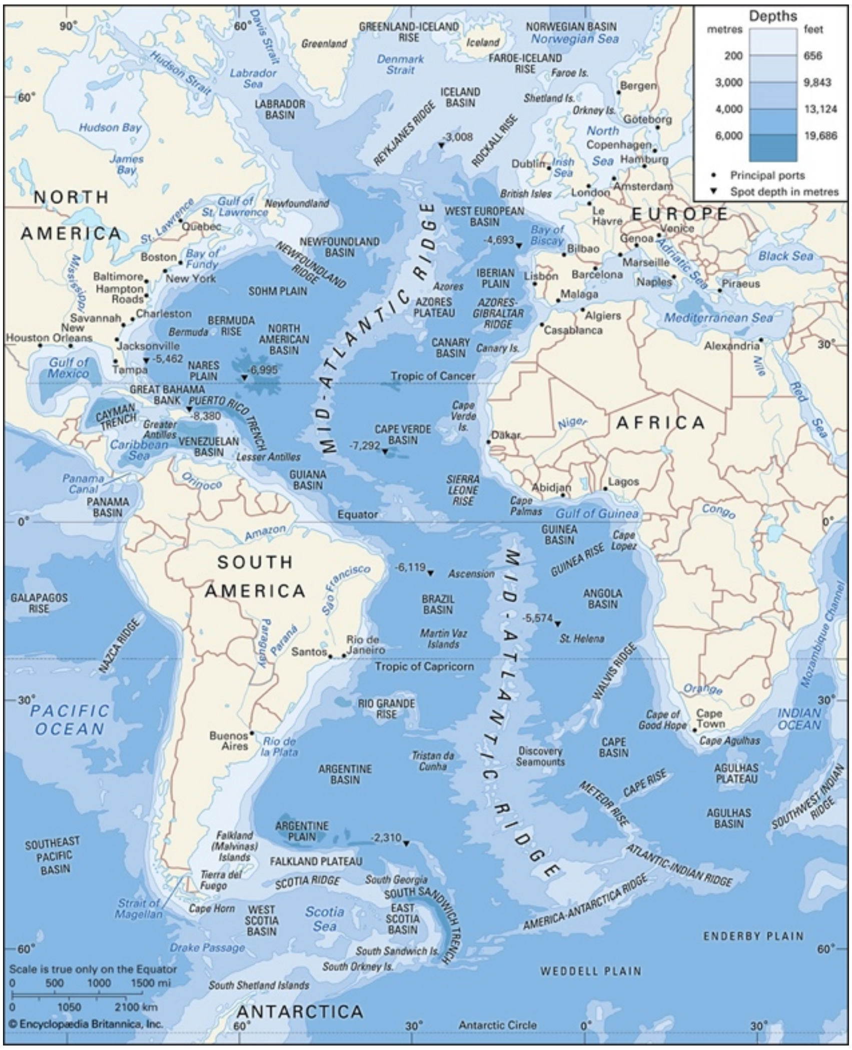

Figure 13A. Basins in the Atlantic which could be used as Level 4 subdivisions of the High Seas (reproduced from <https://www.britannica.com/place/Atlantic-Ocean>

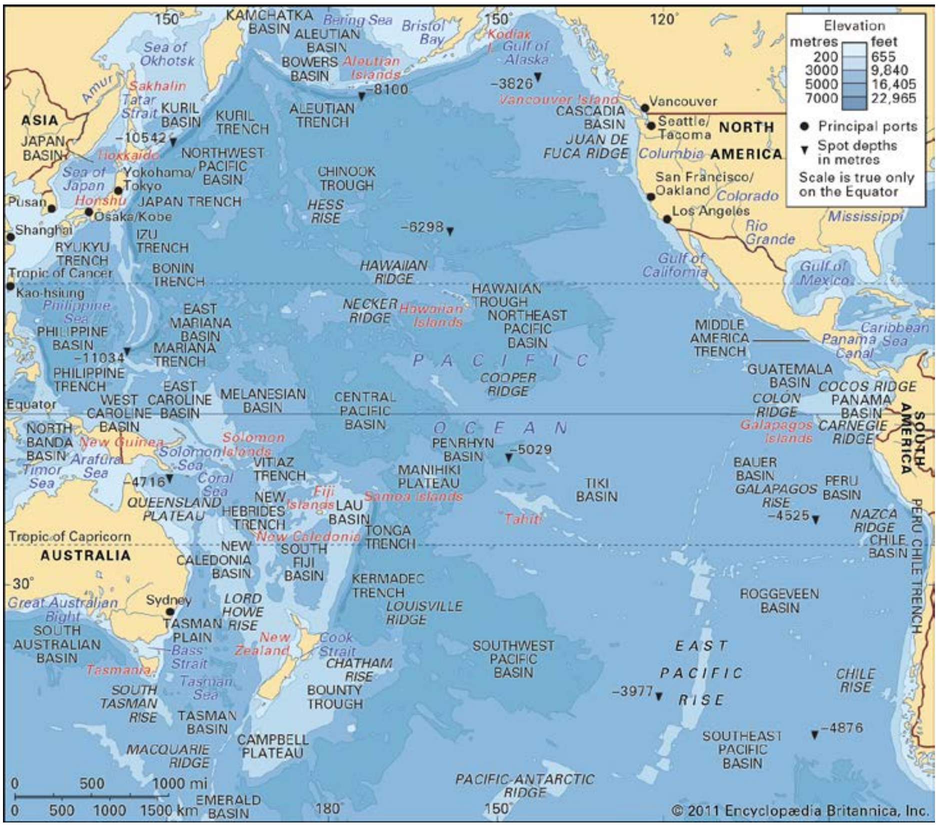

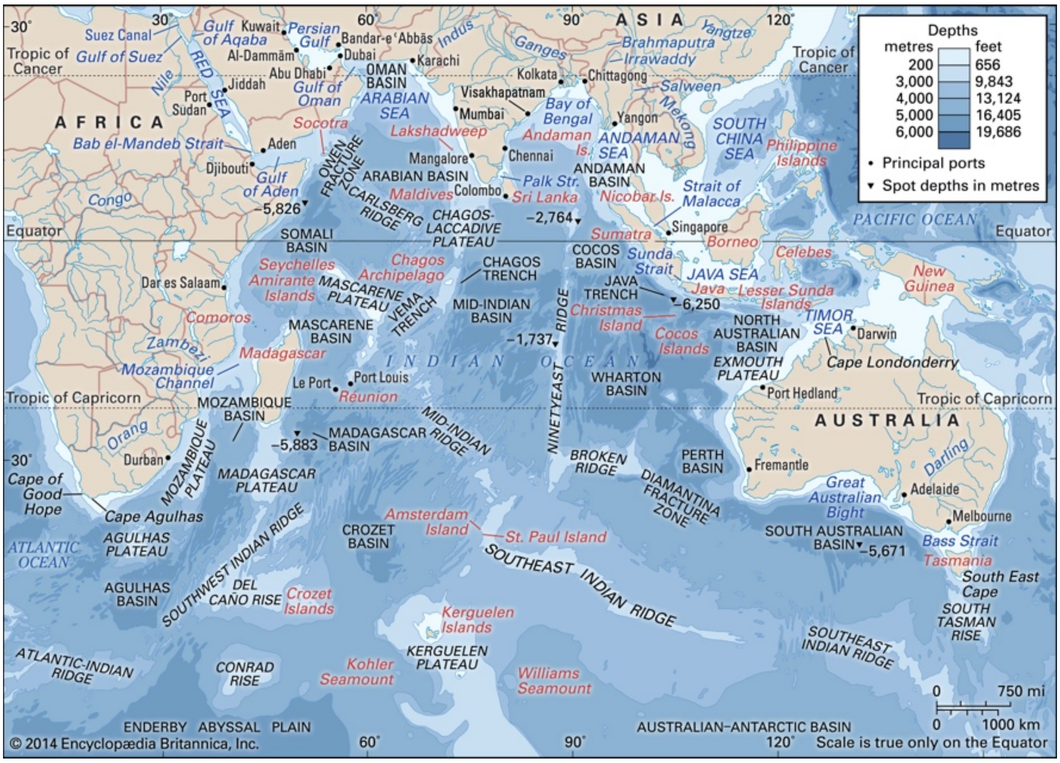

Figure 13B. Basins in the Pacific and Indian oceans, which could be used as Level 4 subdivisions of the High Seas (reproduced from <https://www.britannica.com/place/Pacific-Ocean> and <https://www.britannica.com/place/Indian-Ocean>

Regionalization in the water column

For the pelagic realm, the difference between bathyal, abyssal and hadal is irrelevant to near-surface plankton. The mesopelagic biome was tackled by Sutton et al. (2017) but there, with the same rationale already put forward for the coastal units, preference should be given to a single set of geounits without taking habitats into account. Spalding et al. (2012) indeed produced a coherent regionalization of the pelagic realm, but their units would conflict with those accounting for the benthos. Actually, most biogeographic data regarding plankton originate from oceanographic cruises in which samples are documented with coordinates, therefore a practical solution could be to use only higher level (Level 2) geounits as bounding boxes and point data as the basis for analyses... We acknowledge that our proposed scheme is not fitted for handling occurrences within the water column and its water masses. We still find it useful for recording such occurrences within EEZs (thereby providing information for country checklists) but for the high seas, the only sensible solution would be to define alternative Level 3 and 4 geounits for the water column

References

Allen J.A. (2008). Bivalvia of the deep Atlantic. *Malacologia*, 50(1): 57-173.

Belbin, L., Wallis, E., Hobern, D., & Zerger, A. (2021). The Atlas of Living Australia: History, current state and future directions. Biodiversity Data Journal, 9, e65023 (35 pp.). https://doi.org/10.3897/BDJ.9.e65023

Briggs J.C. (1974). *Marine Zoogeography.* New York: McGraw-Hill, 475 pp.

Briggs J.C. & Bowen B.W. (2012). A realignment of marine biogeographic provinces with particular reference to fish distributions. *Journal of Biogeography*, 39(1): 12-30. https://doi.org/10.1111/j.1365-2699.2011.02613.x

Brummitt R.K., Pando F., Hollis S., & Brummitt N.A. (2001). World Geographic Scheme for Recording Plant Distributions Hunt Institute for Botanical Documentation, Carnegie Mellon University. pp i-xv, 1-102, maps 1-17. Available from http://rs.tdwg.org/wgsrpd/doc/data/, accessed 02-2022

Costello M.J., Tsai P., Wong P.S., Cheung A.K.L., Basher Z., & Chaudhary C. (2017). Marine biogeographic realms and species endemicity. *Nature communications*, 8(1): 1057 (10 pp.). https://doi.org/10.1038/s41467-017-01121-2

Ekman S. (1935). *Tiergeographie des Meeres.* Leipzig: Akademische Verlagsgesellschaft, xii + 542 pp.

Ekman S. (1953). *Zoogeography of the sea.* London: Sidgwick and Jackson, 417 pp.

Emery K.O., Uchupi E., Phillips J., Bowin C., & Mascle J. (1975). Continental margin off western Africa: Angola to Sierra Leone. *AAPG Bulletin*, 59(12): 2209-2265.

Evans K., Bax N., & Smith D. (2017). *Australia State of the Environment 2016: Marine Environment*, Independent Report to the Australian Government Minister for the Environment and Energy. Australian Government, Depart of the Environment and Energy, Canberra, 224 pp. Available from <https://soe.dcceew.gov.au/sites/default/files/2022-05/soe2016-marine-launch_v36march17.pdf>

FAO Coordinating Working Party on Fishery Statistics (2025). The CWP Handbook of Fishery Statistics. Online document, available at <https://www.fao.org/cwp-on-fishery-statistics/handbook/en/>, accessed June 17, 2025.

Flanders Marine Institute. (2019). Maritime Boundaries Geodatabase: Maritime Boundaries and Exclusive Economic Zones (200NM), version 11. Available online at http://www.marineregions.org/. https://doi.org/10.14284/386

Fofonoff P.W., Ruiz G.M., Steves B., Simkanin C., & Carlton J.T. (2018). NEMESIS: National Exotic Marine and Estuarine Species Information System. http://invasions.si.edu/nemesis. Access Date:

Grassle J.F. (2000). The Ocean Biogeographic Information System (OBIS): an on-line, worldwide atlas for accessing, modeling and mapping marine biological data in a multidimensional geographic context. *Oceanography*, 13(3), 5-7.

Harris P.T., Macmillan-Lawler M., Rupp J., & Baker E.K. (2014). Geomorphology of the oceans. *Marine Geology*, 352: 4-24. https://doi.org/10.1016/j.margeo.2014.01.011

Hollis S. & Brummitt R.K. (1992). *World Geographical Scheme for Recording Plant Distributions*. Plant Taxonomic Database Standards no. 2, version 1.0. Hunt Institute for Botanical Documentation, Carnegie Mellon University, Pittsburgh.. ix + 104 pp., 16 maps

Hooker S. B. & McClain C.R. (2000). The calibration and validation of SeaWiFS data. *Progress in Oceanography*, 45(3-4), 427-465.

International Hydrographic Organization (1953). *Limits of oceans and seas*. International Hydrographic Organization, Monte-Carlo, Special publication n§ 28, 38 pp. + erratum.

Jensen H.M., Panagiotidis P. & Reker J. (2017). Delineation of the MSFD Article 4 marine regions and subregions Version 1.0. Technical document of the European Environmental Agency, 21 pp. Available from https://web.archive.org/web/20170605025903/https://www.eea.europa.eu/data-and-maps/data/msfd-regions-and-subregions/technical-document/pdf/download

Kelleher G., Bleakley C., & Wells S. (1995) A Global Representative System of Marine Protected Areas, v.1. Antarctic, Arctic, Mediterranean, Northwest Atlantic, Northeast Atlantic and Baltic. xii+ 219 pp., maps; - v.2. Wider Caribbean, West Africa and South Atlantic, iv+ 93p., maps. - v.3. Central Indian Ocean, Arabian Seas, East Africa and East Asian Seas, iv + 147 pp., maps. - v.4. South Pacific, Northeast Pacific, Northwest Pacific, Southeast Pacific and Australia/New Zealand. , Washington, DC, USA: World Bank.

Kennedy B.R.C. & Rotjan R.D. (2023) Mind the gap: comparing exploration effort with global biodiversity patterns and climate projections to determine ocean areas with greatest exploration needs. *Frontiers in Marine Science*, 10: 1219799 (10 pp.). https://doi.org/10.3389/fmars.2023.1219799

Longhurst A. (1998) *Ecological Geography of the Sea*. Academic Press, San Diego, xv + 542 pp., 22 plates.

Neubauer T., Gofas S., & Harzhauser M. 2025. Biogeographic patterns of modern benthic shallow-water molluscs and the roles of temperature and palaeogeographic legacy. Scientific Reports, 15: 20304 (13 pp.). https://doi.org/10.1038/s41598-025-06473-0

Poore G.C.B. & O'Hara T.D. (2007). Marine biogeography and biodiversity of Australia. Pp. 177-198 in: Connell S.D., and Gillanders B.M. (eds), *Marine Ecology*. Oxford University Press: South Melbourne. https://doi.org/10.1201/9781315373096

Sayre R. G., Wright D. J., Breyer S. P., Butler K. A., Van Graafeiland K., Costello M. J., Harris P.T., Goodin K.L., Guinotte J. M., Basher Z., Kavanaugh M.T., Halpin P.N., Monaco M.E., Cressie N., Aniello P., Frye C.E., Stephens, D. (2017). A three-dimensional mapping of the ocean based on environmental data. *Oceanography*, 30(1): 90-103. https://www.jstor.org/stable/10.2307/24897845

Sherman K. (1991). The Large Marine Ecosystem concept: research and management strategy for living marine resources. *Ecological Applications*, 1(4): 350-360.

Spalding, M. D., Fox, H. E., Allen, G. R., Davidson, N., Ferda¤a, Z. A., Finlayson, M., ... & Robertson, J. (2007). Marine ecoregions of the world: a bioregionalization of coastal and shelf areas. *BioScience*, 57: 573-583. https://doi.org/10.1641/B570707

Spalding M. D., Agostini V. N., Rice J., & Grant S. M. (2012). Pelagic provinces of the world: A biogeographic classification of the world's surface pelagic waters. *Ocean & Coastal Management*, 60: 19-30. https://doi.org/10.1016/j.ocecoaman.2011.12.016

Sutton T.T., Clark M.R., Dunn D.C., Halpin P.N., Rogers A.D., Guinotte J., ... & Heino M. (2017). A global biogeographic classification of the mesopelagic zone. *Deep Sea Research Part I: Oceanographic Research Papers*, 126: 85-102. https://doi.org/10.1016/j.dsr.2017.05.006

Toonen R.J., Bowen B.W., Iacchei M., & Briggs J.C. (2016). Biogeography, Marine. In R.M. Kliman (Ed.), *Encyclopedia of evolutionary biology* (Vol. 1, pp. 166-178). Oxford: Academic Press. https://doi.org/10.1016/B978-0-12-800049-6.00120-7

UNESCO (2009). Global Open Oceans and Deep Seabed (GOODS) ? Biogeographic Classification. Paris, UNESCO-IOC, Technical Series 84, 87 pp. <https://unesdoc.unesco.org/ark:/48223/pf0000182451 >

Watling L., Guinotte J., Clark M.R., & Smith C. R. (2013). A proposed biogeography of the deep ocean floor. *Progress in Oceanography*, 111: 91-112.

Watling L. & Gerken S. (1999) Coastal Marine Bioregions https://web.archive.org/web/19991010011810/http://marine.rutgers.edu:80/OBIS/biogeo/watling.htm [no longer available on the Rutgers website, last accessed there 15 March 2005]

Wu, Z., Li, J., Jin, X., Fang, Y., Shang, J., & Li, S. (2013). Methods and procedures to determine the outer limits of the continental shelf beyond 200 nautical miles. *Acta Oceanologica Sinica*, 32(12): 126-132.

Zhao Q., Basher Z., & Costello M.J. (2020) Mapping near surface global marine ecosystems through cluster analysis of environmental data. *Ecological Research*, 35: 327–342. https://doi.org/10.1111/1440-1703.12060
